# miR-1, miR-10b, miR-155, and miR-191 are novel regulators of BDNF

**DOI:** 10.1007/s00018-014-1628-x

**Published:** 2014-05-08

**Authors:** Kärt Varendi, Anmol Kumar, Mari-Anne Härma, Jaan-Olle Andressoo

**Affiliations:** Institute of Biotechnology, University of Helsinki, 00014 Helsinki, Finland

**Keywords:** Brain-derived neurotrophic factor, MicroRNAs, 3′ untranslated region, Differentiation

## Abstract

Brain-derived neurotrophic factor (BDNF) is a secreted protein of the neurotrophin family that regulates brain development, synaptogenesis, memory and learning, as well as development of peripheral organs, such as angiogenesis in the heart and postnatal growth and repair of skeletal muscle. However, while precise regulation of BDNF levels is an important determinant in defining the biological outcome, the role of microRNAs (miRs) in modulating BDNF expression has not been extensively analyzed. Using in silico approaches, reporter systems, and analysis of endogenous BDNF, we show that miR-1, miR-10b, miR-155, and miR-191 directly repress BDNF through binding to their predicted sites in BDNF 3′UTR. We find that the overexpression of miR-1 and miR-10b suppresses endogenous BDNF protein levels and that silencing endogenous miR-10b increases BDNF mRNA and protein levels. Furthermore, we show that miR-1/206 binding sites within BDNF 3′UTR are used in differentiated myotubes but not in undifferentiated myoblasts. Finally, our data from two cell lines suggest that endogenous miR-1/206 and miR-10 family miRs act cooperatively in suppressing BDNF through their predicted sites in BDNF 3′UTR. In conclusion, our results highlight miR-1, miR-10b, miR-155, and miR-191 as novel regulators of BDNF long and short 3′UTR isoforms, supporting future research in different physiological and pathological contexts.

**Electronic supplementary material:**

The online version of this article (doi:10.1007/s00018-014-1628-x) contains supplementary material, which is available to authorized users.

## Introduction

Brain-derived neurotrophic factor (BDNF) is a member of the neurotrophin family and has important functions in brain development, synaptogenesis, and memory and learning [19, 32, 37, 44, 50]. Disrupted BDNF function has been associated with several nervous system disorders, such as Huntington’s disease [23, 69], depression [8, 11], and anxiety [55], in both humans and rodent models. In addition, BDNF is involved in several physiological processes outside the brain, such as angiogenesis in the heart and skeletal muscles [16], myogenic differentiation [49], and skeletal muscle regeneration [13]. As evident from studies with mice heterozygous for the BDNF gene, 50 % reduction in BDNF protein levels is sufficient to cause notable phenotypic changes [1, 2, 15, 45]. Thus, precise regulation of BDNF levels is important in several developmental and pathological processes, highlighting the importance of understanding the mechanisms that control BDNF expression.

The 3′ untranslated regions (3′UTRs) of mammalian genes contain highly conserved sequences [60] important in the regulation of translational efficiency, polyadenylation, and stability of the mRNA. These functions are mediated by binding to RNA-interacting factors, such as microRNAs (miRs) [6, 48]. miRs are short, non-coding RNA molecules predicted to interact with the transcripts of about 60 % of all mammalian protein-coding genes [7]. miRs bind their target mRNAs through a fully complementary seed sequence of 7–8 nucleotides in their 5′ end and less complementary area in the 3′ end, inducing translational repression and/or mRNA degradation [7, 24].

One way to modulate miR-mediated regulation of gene expression is through the generation of alternative mRNA transcripts that differ in the length of the 3′UTR [58]. Brain-derived neurotrophic factor transcripts contain either short (BDNF-SH, 350 nt) or long 3′UTR (BDNF-L, 2891 nt [27]). In addition to length, secondary structure of the 3′UTR is also known to influence mRNA stability, at least in part by modifying accessibility to miR target sites in the mRNA [29, 67, 68]. In general, miR sites near the ends of the 3′UTR are more effective than sites in the center of the 3′UTR, partly because regions in the middle of the 3′UTR are more likely to be incorporated into hairpin structures, hindering access to miRs [29]. BDNF-L contains a stem-loop structure necessary for the regulation of transcript stability [26], raising the possibility that a secondary structure could determine access to factors like miRs that influence mRNA stability.

Despite the presence of more than ten in silico predicted highly conserved binding sites for different miRs in BDNF 3′UTR (www.targetscan.org, see below) and the physiological and pathological relevance of BDNF levels, to date only a few miRs have been studied in relation to BDNF. Although BDNF regulation by miR-206 has been well established in several studies [42, 47, 54], it has remained controversial which of the three miR-206 sites is functional [42, 47]. The potential effect of another miR-1/206 family member, miR-1, which differs from miR-206 by four nucleotides outside the seed region and has a distinct expression pattern from miR-206, has not been investigated to date. Other studies suggesting miR-BDNF 3′UTR interaction lack evidence that the effect of the putative BDNF-regulating miR on BDNF 3′UTR is direct [25, 46]. Most importantly, the effects of endogenous miRs on putative miR binding sites within BDNF 3′UTR have remained unaddressed altogether. Since 3′UTR length and secondary structure are likely to play an important role in miR-mediated target suppression [29, 58], the fact that all previous studies have exclusively analyzed BDNF 3′UTR fragments [25, 42, 46, 47] further stresses a need for analysis of BDNF 3′UTR-miR interaction in the context of native, full-length 3′UTR.

The objective of the current study was to analyze the regulation of both BDNF-L and BDNF-SH full-length 3′UTR isoforms by ten miRs selected based on evolutionary conservation of their seed sequences within BDNF and their expression within tissues or cell lines with known BDNF function [21, 40, 49, 51, 61, 63]. We assessed the effects of miR-1, miR-10b, miR-15a, miR-16, miR-30a, miR-30b, miR-155, miR-182, miR-191 and miR-195 on full-length BDNF-L and BDNF-SH isoforms and identified miR-1, miR-10b, miR-155, and miR-191 as novel direct regulators of BDNF 3′UTR. In addition, we provide data suggesting direct interaction between BDNF long 3′UTR and endogenous miR-1/206 family miRs in a model system of muscle differentiation, where the regulation of BDNF levels is known to be critical. Finally, our results indicate that the binding sites for miR-1 and miR-10 in BDNF 3′UTR are used synergistically by endogenous miRs in several cell lines.

## Materials and methods

### Plasmids and constructs

Full-length and short BDNF 3′UTR sequences were obtained from BAC clone RP24-149F11 (RPCI-24: Mouse (C57BL/6 J Male) (*Mus musculus*) BAC library; BACPAC Resources, Oakland, CA, USA) using primers in Online resource 1 and inserted downstream of Renilla luciferase gene in pGL4.73[*hRluc*/SV40] vector (E6911, Promega, Madison, WI, USA) using restriction with XbaI. Luciferase constructs containing BDNF 3′UTR with mutations in miR binding sites were generated with inverse PCR from the pGL4.73-BDNF3′UTR using primer pairs indicated in Online resource 1 (mutated nucleotides shown in bold). All constructs were verified by sequencing. Cotransfection with pGL4.13[*luc2*/SV40], encoding for firefly luciferase (E668A, Promega, Madison, WI, USA), was used for normalization in the dual luciferase assay.

### Cell culture

Human embryonic kidney 293 (HEK-293) cells were cultured at 37 °C with 5 % CO_2_ in Dulbecco’s modified Eagle’s medium (DMEM) supplemented with 10 % fetal bovine serum (FBS; SV30160, Thermo Fisher Scientific, Waltham, MA, USA) and 1× Normocin (ant-nr-2, InvivoGen, San Diego, CA, USA).

Human retinal pigment epithelium 19 (ARPE-19, [17]) cells were cultured at 37 °C with 5 % CO_2_ in DMEM/F-12 (1:1) medium containing l-glutamine and 15 mM HEPES (31330, Invitrogen/Thermo Fisher Scientific, Waltham, MA, USA) supplemented with 10 % FBS and 1× Normocin.

Human primary glioblastoma U-87 MG cells were cultured at 37 °C with 5 % CO_2_ in DMEM supplemented with 10 % FBS and 1× Normocin. In all experiments, HEK-293, ARPE-19 and U-87 MG cells were split 1:2 on two consecutive days prior to seeding.

C2C12 mouse skeletal myoblast cells [65] were maintained at subconfluent densities at 37 °C with 5 % CO_2_ in DMEM supplemented with 10 % FBS and 1× Normocin (growth medium, GM). Cells were kept at low density to prevent differentiation. Myogenic differentiation was induced by replacing the growth medium with DMEM supplemented with 2 % horse serum (HS; B15-021, PAA/GE Healthcare Life Sciences, Helsinki, Finland) and 1× Normocin (differentiation medium, DM).

### Luciferase assay

For the luciferase assay, cells were seeded to a 96-well plate at a density of 15,000–20,000 cells/well in a final volume of 100 μl. After 24 h of incubation, 80–90 % confluent cells were transfected with 50 μl Opti-MEM I Reduced Serum Medium, GlutaMAX (51985, Gibco/Thermo Fisher Scientific, Waltham, MA, USA) containing 0.3 μl Lipofectamine 2000 Transfection Reagent (11668, Invitrogen/Thermo Fisher Scientific, Waltham, MA, USA), 100 ng pGL4.73[*hRluc*/SV40] encoding for Renilla luciferase with BDNF 3′UTR (BDNF-L), BDNF short 3′UTR (BDNF-SH) or mutated BDNF 3′UTR cloned downstream of the luciferase coding sequence, 10 ng pGL4.13 encoding for firefly luciferase and 10 nM pre-miRs (where indicated, Applied Biosystems/Thermo Fisher Scientific, Waltham, MA, USA) per well. In experiments where miR binding site mutants were compared to each other, equimolar plasmid quantities were used. Cells were incubated for 3 h and transfection medium was replaced with 100 μl of fresh growth medium. Luciferase activity was measured after 24 h with Dual-Luciferase Reporter Assay System (Promega, Madison, WI, USA) as recommended by the manufacturer. Briefly, cells were lysed with 35 μl of Passive Lysis Buffer per well. Culture plate was incubated for 15 min with shaking at 400 rpm at room temperature. To record luminescence, 100 μl of Luciferase Assay Reagent II was added to 30 μl of lysate for first measurement (firefly luciferase) and 100 μl of Stop & Glo Reagent was added for the second measurement (Renilla luciferase). Renilla/firefly luciferase ratio was used for statistical analysis. Three to four replicate wells were used in each experiment and experiments were repeated 3–5 times.

To assess the utilization of miR binding sites in the BDNF 3′UTR during myogenic differentiation, C2C12 cells were seeded to a 96-well plate at a density of 10,000–15,000 cells/well in a final volume of 100 μl. After 24 h of incubation, 40–50 % confluent cells were transfected with 50 μl OptiMEM containing 0.3 μl Lipofectamine, 100 ng pGL4.73[*hRluc*/SV40] encoding for Renilla luciferase with BDNF 3′UTR or mutated BDNF 3′UTR cloned after the luciferase coding sequence and 10 ng pGL4.13 encoding for firefly luciferase per well. Cells were incubated for 3 h and transfection medium was replaced with 100 μl of fresh medium, either GM (to keep cells growing as undifferentiated myoblasts) or DM (to induce differentiation to myotubes). Luciferase activity in myoblasts was measured after 8 h. DM was replaced every day and luciferase activity in myotubes was measured after 4 days, as described above. Renilla/firefly luciferase ratio was used for statistical analysis. Three to four replicate wells were used in each experiment.

### Transfection with pre-miRs

To quantify endogenous BDNF protein levels after treatment with pre-miRs, ARPE-19 cells and U-87 MG cells were seeded to 6- or 12-well plates and incubated for 24 h to reach 90–100 % confluency. The medium was changed to GM and cells were transfected using OptiMEM containing 5 μl (2.5 μl for 12-well plate) Lipofectamine and 100 nM pre-miRs per well in a final volume of 1 ml (500 μl) for 4 h, then replaced with GM and cultured for 48 h. All experiments were performed with at least two biological repeats.

### Silencing endogenous miRs with anti-miRs

To assess endogenous BDNF expression after silencing endogenous miRs, ARPE-19 and U-87 MG cells were seeded to 12-well plates at 90–100 % confluency. Medium was changed to 250 µl OptiMEM containing 6 µM Endoporter transfection agent, (GeneTools, LLC, Philomath, OR, USA) and 10 nM anti-miRs (Exicon, Vedbaek, Denmark) and cultured for 24 h before substituting with 500 µl GM. Cells were cultured for 48 h before mRNA and protein isolation. All experiments were performed with two biological repeats.

### BDNF enzyme-linked immunosorbent assay (ELISA)

Culture medium from cells treated with pre- or anti-miRs was centrifuged at 1,000 rpm to remove debris and used immediately for ELISA or stored at −80 °C. Cells were lysed with lysis buffer containing 137 mM NaCl, 20 mM Tris–HCl (pH 8.0), 1 % NP40, 10 % glycerol, 1 mM PMSF, 10 μg/ml aprotinin, 1 μg/ml leupeptin and 0.5 mM sodium vanadate. Lysate was centrifuged for 20 min at 13,000 rpm at 4 °C and supernatant was used immediately or stored at −80 °C.

To assess BDNF expression in C2C12 myoblasts and myotubes during differentiation, C2C12 cells were seeded to a six-well plate in a density of 500,000 cells/well in 1 ml of GM. After 24 h, culture medium was isolated from undifferentiated myoblasts and replaced with DM to induce differentiation. DM was changed daily and culture medium from myotubes was isolated on day four. Culture medium was centrifuged at 1,000 rpm and used immediately for BDNF ELISA.

Brain-derived neurotrophic factor ELISA was performed using BDNF *E*_max_ ImmunoAssay System (Promega, Madison, WI, USA) as recommended by the manufacturer. Two replicates from each biological repeat were included in ELISA. Brain-derived neurotrophic factor levels were normalized to total protein concentration using DC Protein Assay (500-0116, Bio-Rad, Helsinki, Finland), as recommended by the manufacturer.

### RNA isolation

ARPE-19 and U-87 MG cells and hippocampi of C57BL/6 J mice were homogenized with TRI Reagent (TR 118, Molecular Research Center, Inc., Cincinnati, OH, USA) and RNA was isolated as recommended by the manufacturer. Briefly, after incubation of the homogenate at room temperature for 5 min, 1-bromo-3-chloropropane (BP151, Molecular Research Center, Inc., Cincinnati, OH, USA) was added and the tubes were shaken vigorously for 15 s. The mix was incubated at room temperature for 5 min and centrifuged at 12,000 × *g* for 15 min at 4 °C. The aqueous phase was transferred to a fresh tube containing isopropanol (59300, Sigma-Aldrich, St. Louis, MO, USA), mixed by vortexing and incubated at room temperature for 10 min. Samples were centrifuged at 12,000 × *g* for 8 min at 4 °C and RNA pellet was washed with 70 % ethanol (Altia Oyi, Helsinki, Finland), followed by centrifugation at 7,500 × *g* for 5 min. Ethanol was then removed and the RNA pellet was allowed to briefly air dry and then dissolved in 30–50 μl H_2_O. The RNA samples were frozen immediately and stored at −80 °C until further processing. RNA quantity was measured with NanoDrop (Thermo Scientific, Waltham, MA, USA). The A260/A280 ratio was 1.78–2.01 and RNA yield was 3.5–9.5 μg (18–40 μg for hippocampus RNA).

### Reverse transcription

RNA samples were treated with Turbo DNA-free DNase treatment and removal reagents, as recommended by the manufacturer (AM1907, Invitrogen/Thermo Fisher Scientific, Waltham, MA, USA), to prevent contamination with genomic DNA. cDNA was synthesized from 150–500 ng of RNA (equal amount of RNA was used within a single experiment) with random hexamer primers in a final volume of 20 μl using Transcriptor First Strand cDNA synthesis kit as recommended by the manufacturer (04896866001, Roche, Basel, Switzerland). Briefly, 2 μl of random hexamer primers was mixed with 11 μl of RNA sample diluted with nuclease-free water, and incubated at 65 °C for 10 min. Then 7 μl of mix containing 4 μl of 5× RT buffer, 2 μl of 100 mM dNTP, 0.5 μl of RNase inhibitor and 0.5 μl of Transcriptor reverse transcriptase was added, mixed gently, and incubated at 25 °C for 10 min, 55 °C for 30 min, and 85 °C for 5 min. No reverse transcriptase control was included in each experiment. cDNA was cooled on ice, diluted 1:10, and stored at −20 °C or used immediately for qPCR.

### Quantitative real-time PCR

Quantitative PCR reaction was performed with the LightCycler 480 real-time PCR system (Roche Diagnostics, Basel, Switzerland) using LightCycler 480 SYBR Green I Master, complemented with 2.5 pmol of primers in the final volume of 10 μl on white 384-well plates sealed with adhesive plate sealer (04729749001, Roche, Basel, Switzerland). An amount of 2.5 μl of the diluted cDNA product was used in each reaction. Oligonucleotide primers (Oligomer Oy, Helsinki, Finland) used for the qPCR reactions are indicated in Online resource 1. No-reverse transcription control and no-template control were included for each experiment. Two or three replicates of each reaction were included in the qPCR runs. The following qPCR program was used: [1] pre-incubation 10 min at 95 °C, [2] amplification 10 s at 95 °C, 15 s at 60 °C, 15 s at 72 °C for 45 cycles, [3] melting curve 5 s at 95 °C, 30 s at 55 °C, continuous acquisition mode at 95 °C with two acquisitions per degree Celsius, and [4] cooling 10 s at 40 °C. The results were analyzed with LightCycler 480 Software Release 1.5.0 SP1 using the Absolute Quantification/2nd Derivative Max calculation. The quantification cycle (C_q_) for the no-template control was 40 (or 0) in all experiments. Beta-actin was used as a reference gene. Results for a biological repeat were discarded when the *C*_q_ value for one or more of the replicates was 40 (or 0) or when the *C*_q_ difference between replicates was >1. For each primer pair, primer efficiencies (the ratio of amplified products if average *C*_q_ difference is one; the ideal efficiency would be two) were determined (Online resource 1). Fold difference to the reference gene was calculated according to the following formulation: FD = (*E*_(GOI)_^−*C*q1(GOI)^ + *E*_(GOI)_^−*C*q2(GOI)^)/(*E*_(ref)_^−*C*q1(ref)^ + *E*_(ref)_^−*C*q2(ref)^), where *E*_GOI_ and *E*_ref_ are the primer efficiencies of the gene of interest (GOI) and reference gene (ref), respectively, and *C*_q1_ and *C*_q2_ are the *C*_q_ values of individual replicates.

### microRNA expression

MicroRNA expression was assessed using TaqMan MicroRNA Assay reactions (Applied Biosystems/Thermo Fisher Scientific, Waltham, MA, USA) according to the manufacturer’s recommendations with minor modifications. Briefly, RNA was isolated as described above. cDNA from 0.3–1 μg RNA was synthesized with TaqMan MicroRNA Reverse Transcription Kit (Applied Biosystems/Thermo Fisher Scientific, Waltham, MA, USA) using Megaplex RT Primers, Rodent Pool A or B (Applied Biosystems/Thermo Fisher Scientific, Waltham, MA, USA) without preamplification in a final volume of 7.5 μl. With the exception of miR-155 and miR-182, all the primers in the MegaPlex Rodent Pool were suitable for the amplification of human microRNAs. For miR-155 and miR-182, 5x RT primers provided by the TaqMan MicroRNA Assay were used for reverse transcription reaction. The cDNA product was diluted 1:30 and 2.5 μl of the diluted cDNA was used for each real-time PCR reaction in a final volume of 10 μl in 384-well plates. Each sample was run in duplicate. microRNA expression from mouse-derived cells was normalized to sno202 and microRNA expression from human-derived cells was normalized to miR-191 [52].

### Statistical analysis

All values are presented as mean ± SEM. Statistical significance level was set at *p* < 0.05. Quantitative PCR data was calculated based on primer efficiencies and analyzed using fold difference compared to reference gene. Statistical analysis was performed using paired or unpaired Student’s *t* test, Mann–Whitney *U* test, or one-way ANOVA followed by Tukey’s HSD (honestly significant difference) or Games–Howell post hoc analysis.

## Results

### Both BDNF 3′UTR isoforms are predicted to contain conserved binding sites for multiple miRs

**Fig. 1:**
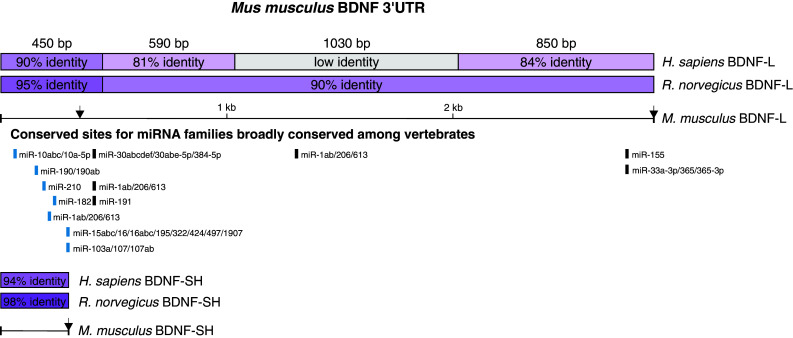
Scheme of long (BDNF-L) and short (BDNF-SH) BDNF 3′UTR isoforms with evolutionarily conserved miR sites predicted by TargetScan. Sites within BDNF-SH are shown in *light blue*. Conservation between mouse, rat, and human BDNF 3′UTR is shown in *purple* *bars*. *Arrows* denote polyadenylation sites

We performed in silico analysis of BDNF 3′UTR-miR interactions using publicly available bioinformatics tool TargetScan (www.targetscan.org) and found that the 3′UTR of BDNF contains evolutionarily conserved seed sequences for multiple miRs (Fig. 1). In addition to miR sites, the overall nucleotide sequence of BDNF 3′UTR is highly conserved across species, especially near both ends of the 3′UTR (Fig. 1), suggesting important biological function of these regions. Most of the predicted miR binding sites fall into the 5′ end of the 3′UTR and the nucleotide sequences near miR sites are highly conserved across vertebrates (Online resource 2). Brain-derived neurotrophic factor transcripts have either a long (BDNF-L, 2891 nt) or short (BDNF-SH, 350 nt) 3′UTR ([27], Fig. 1). Although in silico analysis with different target prediction tools, including TargetScan ([43], http://www.targetscan.org), PITA ([34], http://genie.weizmann.ac.il/pubs/mir07/index.html), miRanda ([33], http://www.microrna.org), PicTar ([38], http://pictar.mdc-berlin.de/) implies that BDNF 3′UTR can be regulated by a multitude of miRs (Online resource 3), to date only a few BDNF 3′UTR-miR interactions have been investigated, and in these, relatively short BDNF 3′UTR fragments have been used [25, 42, 46, 47]. Since there is potential for several miRs to bind BDNF 3′UTR, we set out to systematically investigate the role of ten miRs with conserved putative binding sites in BDNF 3′UTR isoforms in the regulation of BDNF expression. miRs of interest were selected based on the conservation of their binding site within BDNF 3′UTR, co-expression with BDNF in cells and tissues where BDNF is known to have important roles, and/or their overall expression level (Online resource 2, microRNA.org, [20, 22, 31, 40]). We used full-length BDNF-L and BDNF-SH sequences to better preserve the potential effects that secondary structure and nucleotide context may have on miR binding.

### Analysis of BDNF 3′UTR isoforms for miR regulation

**Fig. 2:**
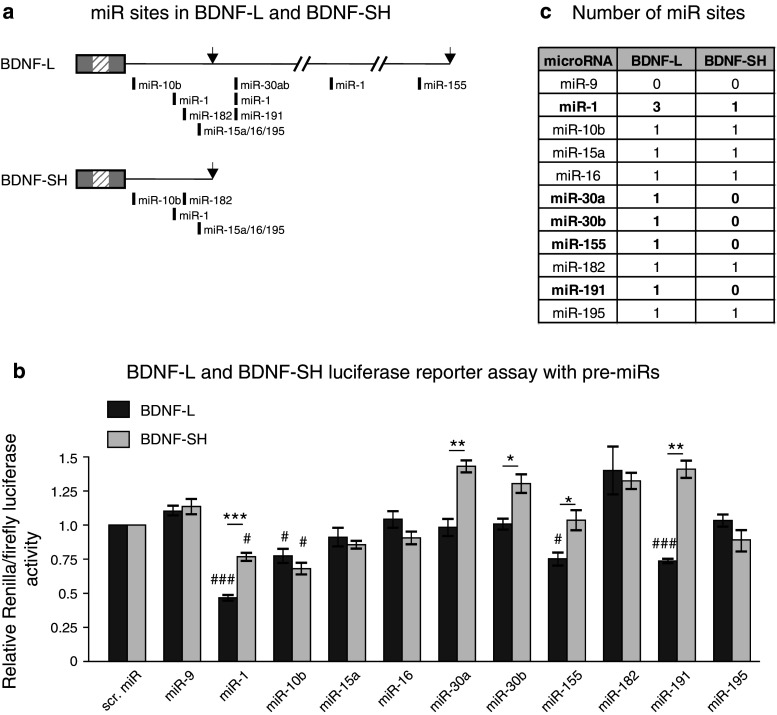
Several of the predicted miRs regulate BDNF 3′UTR. **a** Scheme of miR sites within BDNF-L and BDNF-SH. Only miRs included in the current study are shown. *Arrows* denote polyadenylation sites. **b** Luciferase activity of reporter constructs containing either BDNF-L or BDNF-SH, co-transfected with pre-miRs. *Dark grey bars* show inhibition of BDNF-L by miRs and *light grey bars* show inhibition of BDNF-SH by miRs. *#* indicates difference from treatment with negative control and * indicates differences between the extent of miR inhibition between BDNF-L and BDNF-SH. *n* = 3–8, #*p* < 0.05, ###*p* < 0.001, *p < 0.05, **p < 0.01, ***p < 0.001. **c** Number of miR sites in BDNF-L and BDNF-SH predicted by TargetScan. miRs that have a different number of predicted binding sites within BDNF-L and BDNF-SH are shown in *bold*

We asked whether the in silico predicted miRs (Fig. 1) regulate protein synthesis from a transcript containing full-length BDNF-L. For that, we transfected human embryonic kidney 293 (HEK-293) cells with precursors for ten miRs predicted by four different target prediction tools (Online resource 3) to bind BDNF-L (miR-1, miR-10b, miR-15a, miR-16, miR-30a, miR-30b, miR-155, miR-182, miR-191, and miR-195, Fig. 2a). Scrambled pre-miR (scr. miR) and precursor for miR-9 that do not have a strongly conserved predicted binding site in BDNF-L were used as negative controls. We found that miR-1, which has three binding sites in BDNF-L (Fig. 2a), reduces luciferase signal most efficiently (Fig. 2b). Of the other miRs, miR-10b, miR-155, and miR-191 significantly inhibited luciferase signal compared to the scrambled miR control. Unlike in previous studies, which utilized fragments of BDNF 3′UTR in the same reporter assay, we found no significant effect for miR-30a or miR-195 (Fig. 2b).

In parallel, we tested the ability of the same miRs to inhibit protein synthesis from transcript containing BDNF-SH, which contains binding sites for six miRs out of the ten assessed in BDNF-L (Fig. 2a, c). We found that miR-1 and miR-10b significantly reduced luciferase activity. Of the three miR-1 sites present in BDNF-L, only the first is located within BDNF-SH (Fig. 2a, c). If miR-1 sites act additively to regulate BDNF levels, miR-1 is expected to have a milder effect on a reporter construct carrying BDNF-SH compared to BDNF-L. Matching the prediction, we found a statistically significant difference in the extent of luciferase signal reduction by miR-1 on BDNF-SH compared to BDNF-L (Fig. 2b). Similarly, unlike BDNF-L, BDNF-SH lacks binding sites for miR-155 and miR-191. As expected, miR-155 and miR-191 had no effect on BDNF-SH, while they had a significant effect on BDNF-L (Fig. 2b). Thus, we found a statistically significant difference in the extent of luciferase signal reduction between BDNF-L and BDNF-SH by miR-1, miR-155 and miR-191 that have a different number of predicted binding sites in the long and short BDNF 3′UTR isoforms. In contrast, there was no difference in luciferase activity after treatment with miR-10b that has a single binding site predicted to regulate both BDNF-L and BDNF-SH (Fig. 2a–c). These data suggest that miR-1, miR-10b, miR-155, and miR-191 are novel regulators of BDNF 3′UTR and that a subset of them are able to differentially regulate expression from transcripts containing either short or long 3′UTR isoform.

### Regulation of endogenous BDNF levels by miRs

**Fig. 3:**
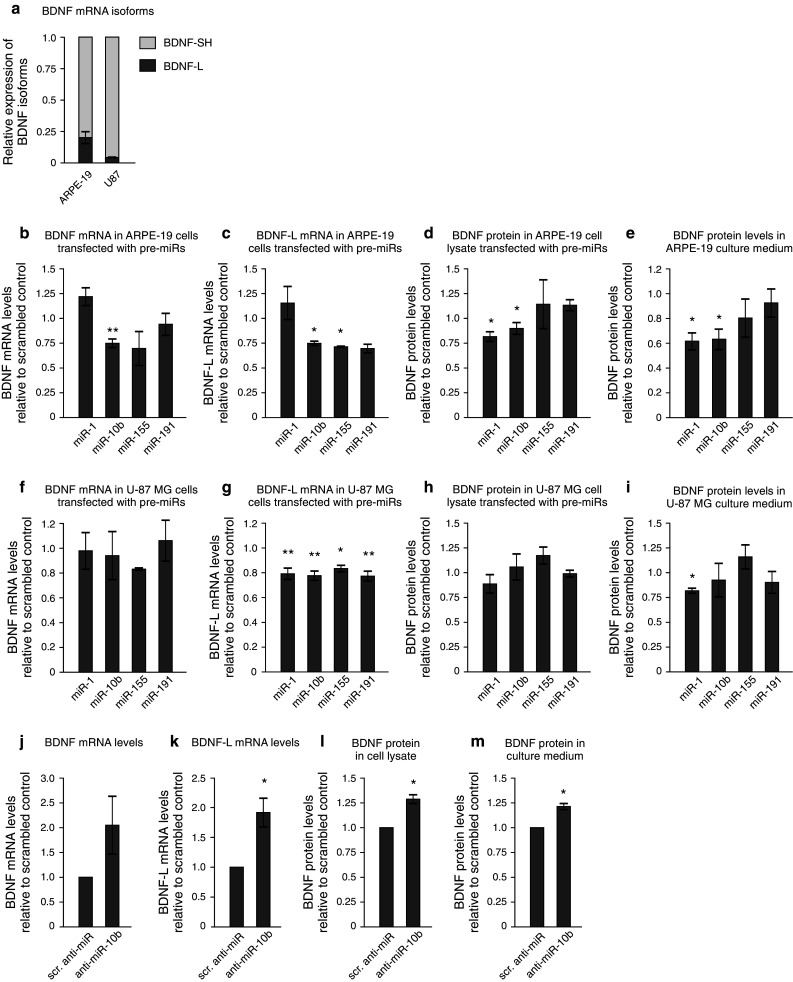
Regulation of endogenous BDNF levels by pre- and anti-miRs. **a** Expression of BDNF-L and BDNF-SH mRNA isoforms in ARPE-19 and U-87 MG cells. Endogenous BDNF levels in ARPE-19 (**b–e**) and U-87 MG **(f–i**) cells transfected with pre-miRs, relative to scrambled control. **j–m** Endogenous BDNF levels in ARPE-19 cells transfected with anti-miRs, relative to scrambled control. **b, f, j** Total BDNF mRNA levels, normalized to β-actin. **c, g, k** mRNA levels of BDNF transcripts containing the long 3′UTR (BDNF-L), normalized to β-actin. **d, h, l** BDNF protein levels in whole cell lysate, normalized to total protein content. **e, i, m** BDNF protein levels in culture medium, normalized to total protein content. *n* = 3.* Asterisks* show difference from negative control, *p < 0.05, **p < 0.01, ***p < 0.001. *Error bars* denote mean ± SEM

Next, we asked if the miRs that regulate BDNF 3′UTR in a reporter assay can also regulate endogenous BDNF. Towards that end, we made use of human retinal pigment epithelial (ARPE-19) cells and human glioblastoma cell-line U-87 MG that secrete a detectable amount of endogenous BDNF. Relative expression of BDNF and BDNF-regulated miRs in different cell lines used in this study is shown in Online resource 4a and 5. We found that approximately 80 % of BDNF mRNA transcripts in ARPE-19 cells and 95 % of BDNF transcripts in U-87 MG cells are expressed as short 3′UTR isoforms (Fig. 3a), suggesting that miR-155 and miR-191, which only have a binding site within BDNF-L, should be unable to regulate the majority of BDNF transcripts in these cells (Fig. 2a). We transfected ARPE-19 cells with precursors for miR-1, miR-10b, miR-155, and miR-191, and measured BDNF mRNA levels with primers recognizing total BDNF and BDNF transcript carrying the long 3′UTR (BDNF-L). miR-10b significantly decreased both total BDNF and BDNF-L levels, while miR-155 and miR-191 only reduced BDNF-L expression, as expected (Fig. 3b–c). We then determined BDNF protein levels in the cell lysate and culture medium by ELISA. We found that miR-1 and miR-10b effectively suppressed endogenous BDNF synthesis, while miR-155 and miR-191 did not significantly reduce BDNF levels (Fig. 3d–e). Brain-derived neurotrophic factor levels in the culture medium were decreased more compared to the whole cell lysate, indicating that in addition to BDNF, miR-1 and miR-10b could regulate the expression of proteins involved in BDNF secretion. Surprisingly, although BDNF mRNA levels were increased by transfection with miR-1, protein levels were decreased (Fig. 3b–e), suggesting that the mechanism by which miR-1 suppresses BDNF may be different from that of other miRs and may involve other factors that regulate BDNF expression. Taken together, these data suggest that miR-1 and miR-10b inhibit endogenous BDNF levels but do not exclude the ability of miR-155 and miR-191 to do the same in cells expressing BDNF transcript with the long 3′UTR isoform.

Since BDNF has multiple functions in the central nervous system, we also tested pre-miRs in U-87 MG glioblastoma cells that originate from the CNS. However, only 5 % of BDNF transcripts in these cells carry the long 3′UTR isoform (Fig. 3a). We found that while total BDNF levels were not reduced after treatment with any of the pre-miRs, the expression of BDNF transcripts carrying the long 3′UTR was significantly decreased by about 20 % by all the pre-miRs tested (Fig. 3f–g). Interestingly, although miR-1 and miR-10b have a binding site within BDNF-SH and should therefore be able to regulate the expression of all BDNF transcripts, they seem to be effective only in suppressing BDNF-L in U-87 MG cells (Fig. 3f–g). Consistent with our finding that miRs only regulate a marginal subpopulation of BDNF transcripts, resulting in about 20 % decrease in BDNF-L levels, BDNF protein levels in U-87 MG cells were not suppressed by transfection with pre-miRs (Fig. 3h–i).

Finally, we transfected ARPE-19 cells with anti-miR-10 and found that silencing endogenous miR-10b significantly increased BDNF mRNA and protein levels (Fig. 3j–m). We were unable to address the effect of miR-1 in ARPE-19 cells, since it is not expressed in this cell line (Online resource 5). In summary, our results suggest that miR-1, miR-10b, miR-155, and miR-191 are able to regulate BDNF expression, although the effect may vary depending on the cell line.

### miR-1, miR-10b, miR-155, and miR-191 regulate BDNF 3′UTR directly through their predicted sites

**Fig. 4:**
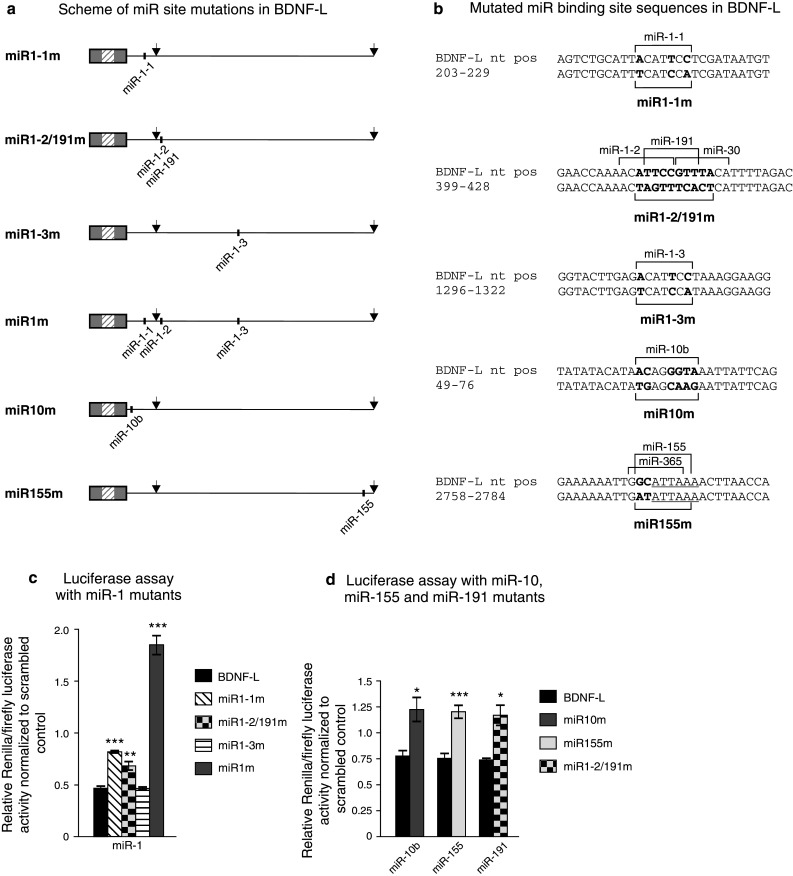
Regulation of luciferase signal by miRs is specific to the predicted miR binding sites. **a** Schemes of miR site mutations in BDNF-L. Mutated sites are shown for each BDNF-L mutant. *Arrows* denote polyadenylation sites. **b** miR binding site mutations in BDNF-L. BDNF 3′UTR sequences complementary to miR seed sites are indicated with *brackets*. Mutated nucleotides are shown in *bold*. **c** Mutating miR-1 predicted binding sites in BDNF-L prevents repression by miR-1 in a luciferase reporter assay in HEK-293 cells. *n* = 3. **d** Mutating miR-10b, miR-155, and miR-191 predicted binding sites prevents repression by the respective miRs in a luciferase reporter assay in HEK-293 cells. *n* = 3. **c, d** Reporter expression is shown relative to scrambled pre-miR. *Error bars* denote mean ± SEM. *Asterisks* show difference from BDNF-L; *p < 0.05, **p < 0.01, ***p < 0.001

Quantifying endogenous BDNF expression after treatment with pre- or anti-miRs does not allow to distinguish whether miRs regulate BDNF levels directly or indirectly. To investigate the interaction between the miRs identified to suppress BDNF-L (Fig. 2b) and their concomitant binding sites within the BDNF 3′UTR, we created constructs harboring mutations in one or several miR binding sites in BDNF-L (Fig. 4a–b). Since miR-1 has three predicted sites, we generated mutant constructs for each of the sites individually (miR1-1m, miR1-2/191m, miR1-3m) and a triple mutant with all three binding sites mutated (miR1m). It should be noted that the second binding site of miR-1 (miR-1-2) partly overlaps with miR-191 binding site and therefore both sites were mutated in miR-1-2 mutant (Fig. 4a–b). We co-transfected mutant constructs with the corresponding pre-miRs into HEK-293 cells as above and assessed the relative extent of inhibition. In accordance with analysis of BDNF-L vs. BDNF-SH (Fig. 2b), mutating the first site (miR1-1m), which is also present within BDNF-SH, reduced suppression by miR-1 most effectively compared to mutating the other miR-1 sites (Fig. 4c), suggesting that miR-1-1 site is more important in regulating BDNF 3′UTR than miR-1-2 and miR-1-3 sites. Furthermore, mutating the third miR-1 site (miR1-3m) did not change 3′UTR inhibition by exogenous miR-1 (Fig. 4c), suggesting that this site may not be used by miR-1 for suppression of BDNF 3′UTR. Mutating miR-10b (miR10m), miR-155 (miR155m), and miR-191 (miR1-2/191m) sites effectively abolished the inhibitory effect of the corresponding exogenous miRs (Fig. 4d), suggesting that these miRs also directly inhibit protein synthesis by binding to the predicted site in the BDNF 3′UTR.

### miR-1/206 sites in BDNF 3′UTR are used by endogenous miRs in differentiated but not in undifferentiated muscle cells

**Fig. 5:**
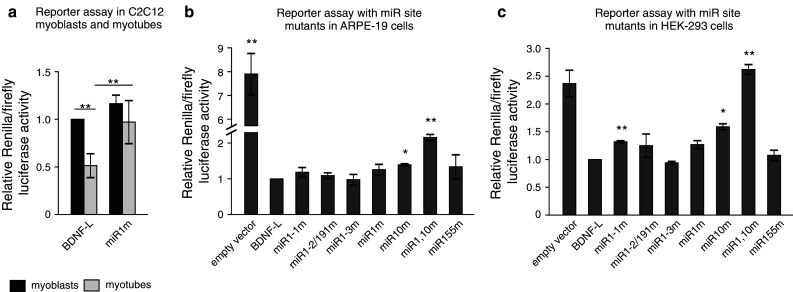
Mutating miR-1 and miR-10 sites abolishes suppression of BDNF-L by endogenous miRs in a reporter assay. **a** Reporter activity in C2C12 cells before and after differentiation with constructs containing either wild-type BDNF-L or BDNF-L miR site mutants. *n* = 7. **b, c** Luciferase activity of reporter constructs containing wild-type BDNF-L or BDNF-L miR site mutants. *Asterisks* show difference from BDNF-L. *n* = 3–4. **b** Luciferase activity in ARPE-19 cells. **c** Luciferase activity in HEK-293 cells. *n* = 3–5. *Error bars* denote mean ± SEM. *p < 0.05, **p < 0.01, ***p < 0.001

Regulation of BDNF expression has been shown to be required for postnatal growth and repair of skeletal muscle in vivo [13]. To gain further insight into the physiological relevance of miR sites identified to regulate BDNF 3′UTR, we extended our analysis to a model of muscle differentiation, the mouse myoblast C2C12 cells, where reduction in BDNF levels is believed to be required to allow myotube generation from undifferentiated myoblasts [47]. miR-206, a miR-1/206 microRNA family member that has identical seed sequence to miR-1 but differs from miR-1 by four nucleotides (Online resource 4b), is believed to be involved in BDNF downregulation in C2C12 cells [47]. However, it is unknown whether the predicted miR-1/206 sites are the sites used by endogenous miR-1 and/or miR-206 during muscle differentiation. Furthermore, it has remained unclear if BDNF suppression by miR-1/206 is used in myoblasts to induce differentiation, in myotubes to maintain differentiation, or both. To gain further insight into these questions, we first confirmed earlier findings that BDNF is downregulated with concurrent upregulation of miR-1 and miR-206 upon myogenic differentiation of muscle cells (Online resource 4c–e; [12, 35, 47, 49]). Then we analyzed the effect of miR-1/206 site mutations on reporter construct expression in myoblasts and myotubes. We found that BDNF-L was repressed in differentiated myotubes by about 50 %, while miR1m mutant construct was not (Fig. 5a). Furthermore, although both miR-1 and miR-206 are expressed in myoblasts (Online resource 4e, 5), there was no difference in the expression of BDNF-L and miR1m containing reporters in those cells (Fig. 5a). This suggests that interaction between BDNF 3′UTR and miR-1/206 family miRs is utilized in C2C12 myotubes to maintain differentiation by BDNF repression but not to induce myogenic differentiation.

### Endogenous miRs regulate BDNF 3′UTR in different cell lines through the predicted sites

Next we turned to cell lines where interaction between BDNF 3′UTR and endogenous miRs has not been addressed before. We determined the endogenous miR expression levels in human embryonic kidney cell line HEK-293 and human retinal pigment epithelial cell line ARPE-19. We found that miR-1 was not expressed in ARPE-19 cells and miR-155 was not expressed in HEK-293 cells, while the other miRs were expressed but their relative levels were different between HEK-293 and ARPE-19 cells (Online resource 5). To assess the interaction between BDNF 3′UTR and endogenous miRs, we transfected HEK-293 and ARPE-19 cells with luciferase assay reporter containing native BDNF-L or constructs harboring various miR site mutants: mutations for each individual miR-1 site (miR1-1m, miR1-2/191m, miR1-3m), all three miR-1 binding sites (miR1m), miR-10b site (miR10m), miR-155 site (miR155m) and a quadruple mutant for three miR-1 sites and miR-10b site (miR1,10m). As indicated above, miR-191 site was mutated in miR1-2/191m mutant construct (Fig. 4b). We found that mutating miR-10b site significantly derepressed luciferase construct in both cell lines (Fig. 5b–c). Consistent with our findings with miR-1 transfection (Fig. 4c), we found that mutating the miR-1-1 site, but not the other miR-1 sites increased luciferase signal in HEK-293 cells (Fig. 5c). In ARPE-19 cells, luciferase signal obtained with the quadruple mutant miR1,10m was increased by approximately two-fold compared to wild-type BDNF-L. However, in HEK-293 cells, mutating all three sites for miR-1 and the single miR-10b site (miR1,10m) derepressed luciferase activity to the level comparable to the empty luciferase vector carrying SV40 late 3′UTR (Fig. 5c), which is in essence devoid of binding sites for strongly conserved miRs (Online resource 6). These results indicate that miR-1 and miR-10 sites within BDNF 3′UTR are used synergistically by endogenous miR-1/206 and miR-10 family miRs to repress BDNF 3′UTR, and at least in HEK-293 cells miR-1/206 and miR-10 sites are responsible for the majority of BDNF 3′UTR repression.

## Discussion

Here we present the first systematic functional analysis of nine evolutionarily conserved miR recognition sites in BDNF 3′UTR. Unlike previous studies, we have analyzed full-length sequences of both BDNF 3′UTR isoforms, BDNF-L (2891 nt) and BDNF-SH (350 nt). We identify miR-1, miR-10b, miR-155, and miR-191 as novel regulators of BDNF 3′UTR and show that BDNF-L and BDNF-SH can be differentially regulated by a subset of miRs. Using mutated constructs we show that the interaction between the identified miRs and BDNF 3′UTR is direct. Our results suggest that miR-1/206 sites within BDNF 3′UTR are used in C2C12 myotubes to maintain differentiation rather than in myoblasts to induce differentiation. Finally, we show that the predicted binding sites for miR-1/206 and miR-10 family miRs are used in a synergistic manner to repress BDNF 3′UTR in ARPE-19 and HEK-293 cells.

The potential physiological importance of BDNF regulation by miRs has been suggested by a study where Dicer, an enzyme required for miR maturation, was conditionally deleted from the forebrain. Prior to the onset of neurodegeneration, BDNF levels in such animals were increased with concomitant functional changes in the CNS [36]. Recently, the conclusion was confirmed by Lee et al., who demonstrated BDNF regulation by miR-206 in the brain [42]. In addition, other studies have addressed the potential interaction between BDNF 3′UTR and specific miRs. Interestingly, miR-30a and miR-195 did not have an effect in our study (Fig. 2b) while previous results have suggested that these miRs regulate BDNF 3′UTR in a reporter assay [46]. These different results can at least partly be explained by the use of 3′UTR fragments in the reporter assay of the previous study, which may affect the accessibility of miRs to their binding site in the target mRNA. It has been shown that miR sites residing near the two ends of long 3′UTRs are generally more effective than those near the center [29]. Thus, a site normally unavailable in the native 3′UTR may become more accessible if only a small region of the 3′UTR is analyzed in reporter assay and in some cases, the situation may be the other way around. miR-191 has been shown not to inhibit BDNF 3′UTR [46], whereas our results clearly show that miR-191 does in fact directly inhibit BDNF 3′UTR via the predicted site (Fig. 2b, 4d).

Further illustrating the potential problems stemming from analysis of fragments rather than full-length 3′UTRs are the conflicting results of studies by Miura et al. and Lee et al. [42, 47], where the first study showed that of three miR-206 sites within BDNF 3′UTR, the first two and not the third are functional, while the other study reached the opposite conclusion. Although both studies analyzed fragments of BDNF 3′UTR, the size of fragments used in [47] was longer than those studied in [42] and thus resembled the full-length BDNF 3′UTR better. Our analysis of full-length BDNF 3′UTR interaction with miR-1, another family member of miR-1/206 family, supports the conclusion of (Fig. 2b, 4c, [47]). Our data on miR-1 also confirms the in silico prediction that miR-1 and miR-206 share the binding sites on BDNF 3′UTR (Fig. 1). However, it is important to keep in mind that miR-1 and miR-206 differ by four nucleotides outside the seed sequence (Online resource 4b). Therefore, it would be interesting to analyze miR-206 and BDNF 3′UTR interaction in the context of full-length 3′UTR.

We also found that endogenous miR-1/206 and miR-10 family miRs act cooperatively to repress BDNF expression through its 3′UTR in two cell lines of different origin (Fig. 5b–c). This is consistent with the notion from previous reports that multiple miR sites within a 3′UTR can act in a synergistic manner, especially if the sites are located close together [29, 56, 57]. In line with this, recent experimental evidence shows that noncoding pseudogenes can bind to and compete for the same combination of miRs as their ancestral gene [53], suggesting that there has been evolutionary pressure on pseudogenes to preserve the same miR binding site pattern or “miR code” that is used to regulate protein synthesis from the ancestral transcript. Even though the expression levels of pseudogenes are low, they seem to be sufficient to derepress endogenous parent gene expression in case their 3′UTR contains binding sites for multiple miRs that act in synergy. Our data imply that regulation of BDNF by its 3′UTR is important in tissues where miR-1/206 and miR-10 family miRs are co-expressed. In addition, although mutating miR-155 and miR-191 sites had no effect on reporter activity in HEK-293 and ARPE-19 cells, it cannot be excluded that BDNF 3′UTR interacts with miR-155 or miR-191 in other tissues or cell types.

Although some studies have suggested that inhibition by miRs depends mostly on miR expression levels [18, 28], others suggest that additional factors such as specific nucleotide composition [53, 57], or the number of target genes [4] may be more important in defining the repressive activity of miRs. Our results indicate that endogenous miR expression levels do not solely determine the inhibition efficiency. In assays utilizing reporter constructs and pre-miR transient transfection, miR-1 through its sites 1 and 2, miR-10b and miR-191 all suppress BDNF 3′UTR (Fig. 2b, 4c–d). However, despite the presence of relatively high levels of endogenous miR-191 compared to endogenous miR-1 and miR-10b in both HEK-293 and ARPE-19 cells (Online resource 5), mutating miR-191 site did not lead to an increase in luciferase signal, whereas mutating miR-10b site or miR-1 sites together with miR-10b site had a clear effect (Fig. 5b–c). Thus, results suggesting direct interaction between miR and 3′UTR in transient transfection assays do not allow conclusions on the interaction between endogenous miR and the corresponding site within the 3′UTR, even if the endogenous miR expression levels are high, underlining the need to test each case separately.

While the role of miR-1/206 family member miR-206 in the regulation of BDNF levels is well established [42, 47, 54], the interaction between BDNF 3′UTR and miR-1 has not been investigated. Although miR-1 and miR-206 have identical seed sequences, the mature miR-1 differs from miR-206 by four nucleotides (Online resource 4b). miR-1 is encoded by two distinct genomic locations in both mouse and human genomes and displays partially different expression pattern from miR-206. For example, both miR-1 and miR-206 are expressed in high levels in the skeletal muscle [12, 35, 39, 59, 68], but unlike miR-206, miR-1 levels are high in the heart [40]. Since BDNF is known to be required for heart angiogenesis [16], it would be interesting to assess the role of miR-1 and BDNF interaction in heart development. Moreover, appropriate BDNF levels are important for adjusting the number of dopaminergic neurons in the substantia nigra midbrain [5] and regulation of BDNF expression in the hippocampus is important in learning and memory (see [64]). miR-1 is expressed within the midbrain [40], hippocampus (Online resource 5), and in several other neuron populations within the central nervous system [30]. Taken together, there is potential for miR-1-mediated regulation of BDNF levels during development, warranting further investigation.

While miR-191 is widely expressed in different tissues and cell types, miR-10b and miR-155 have a more restricted expression pattern [40]. For example, one of the few expression sites of miR-10b is the skin [40], whereas expression of BDNF in the skin is required for its proper sensory innervation [21]. miR-155, on the other hand, is robustly upregulated after the activation of immune cells, including microglia [9], whereas BDNF expression by microglia has been implicated in the induction of neuropathic pain [14, 66].

Our results also suggest that selected miRs are able to differentially regulate BDNF transcripts with either long or short 3′UTR isoform. Indeed, the considerably shorter half-life of BDNF mRNAs containing the long 3′UTR compared to the short 3′UTR [10] may at least partly be a consequence of miR regulation. Furthermore, the relative abundance of BDNF 3′UTR isoforms varies during developmental processes, for example, in muscle differentiation [47], and in different brain areas, such as the cortex, hippocampus and cerebellum [3, 62], suggesting that the long and short 3′UTR isoforms have different biological functions. In line with this, it was recently shown that in response to neuronal activation, BDNF protein synthesis in hippocampal neurons is rapidly initiated from transcripts containing the long 3′UTR, while expression from transcripts containing the short 3′UTR maintains basal BDNF levels [41].

Overall, co-expression of BDNF and miR-1/miR-10b/miR-155/miR-191 in various cell types and tissues suggests potential relevance of the interaction between miRs and BDNF 3′UTR isoforms in different physiological and pathological processes, which remain a target of future studies addressing post-transcriptional BDNF regulation.

### Electronic supplementary material

Below is the link to the electronic supplementary material.

Supplementary material 1 (PDF 74 kb)

Supplementary material 2 (PDF 12 kb)

Supplementary material 3 (PDF 568 kb)

Supplementary material 4 (PDF 399 kb)

Supplementary material 5 (PDF 1,436 kb)

Supplementary material 6 (PDF 63 kb)

Supplementary material 7 (PDF 68 kb)

## References

[1] Abidin I, Eysel UT, Lessmann V, Mittmann T (2008). Impaired GABAergic inhibition in the visual cortex of brain-derived neurotrophic factor heterozygous knockout mice. J Physiol.

[2] Abidin I, Kohler T, Weiler E, Zoidl G, Eysel UT, Lessmann V, Mittmann T (2006). Reduced presynaptic efficiency of excitatory synaptic transmission impairs LTP in the visual cortex of BDNF-heterozygous mice. Eur J Neurosci.

[3] An JJ, Gharami K, Liao GY, Woo NH, Lau AG, Vanevski F, Torre ER, Jones KR, Feng Y, Lu B, Xu B (2008). Distinct role of long 3′ UTR BDNF mRNA in spine morphology and synaptic plasticity in hippocampal neurons. Cell.

[4] Arvey A, Larsson E, Sander C, Leslie CS, Marks DS (2010). Target mRNA abundance dilutes microRNA and siRNA activity. Mol Syst Biol.

[5] Baquet ZC, Bickford PC, Jones KR (2005). Brain-derived neurotrophic factor is required for the establishment of the proper number of dopaminergic neurons in the substantia nigra pars compacta. J Neurosci.

[6] Barrett LW, Fletcher S, Wilton SD (2012). Regulation of eukaryotic gene expression by the untranslated gene regions and other non-coding elements. Cell Mol Life Sci.

[7] Bartel DP (2004). MicroRNAs: genomics, biogenesis, mechanism, and function. Cell.

[8] Calabrese F, Molteni R, Maj PF, Cattaneo A, Gennarelli M, Racagni G, Riva MA (2007). Chronic duloxetine treatment induces specific changes in the expression of BDNF transcripts and in the subcellular localization of the neurotrophin protein. Neuropsychopharmacology.

[9] Cardoso AL, Guedes JR, Pereira de Almeida L, Pedroso de Lima MC (2012). miR-155 modulates microglia-mediated immune response by down-regulating SOCS-1 and promoting cytokine and nitric oxide production. Immunology.

[10] Castren E, Berninger B, Leingartner A, Lindholm D (1998). Regulation of brain-derived neurotrophic factor mRNA levels in hippocampus by neuronal activity. Prog Brain Res.

[11] Chan JP, Unger TJ, Byrnes J, Rios M (2006). Examination of behavioral deficits triggered by targeting Bdnf in fetal or postnatal brains of mice. Neuroscience.

[12] Chen JF, Mandel EM, Thomson JM, Wu Q, Callis TE, Hammond SM, Conlon FL, Wang DZ (2006). The role of microRNA-1 and microRNA-133 in skeletal muscle proliferation and differentiation. Nat Genet.

[13] Clow C, Jasmin BJ (2010). Brain-derived neurotrophic factor regulates satellite cell differentiation and skeletal muscle regeneration. Mol Biol Cell.

[14] Coull JA, Beggs S, Boudreau D, Boivin D, Tsuda M, Inoue K, Gravel C, Salter MW, De Koninck Y (2005). BDNF from microglia causes the shift in neuronal anion gradient underlying neuropathic pain. Nature.

[15] Dluzen DE, Anderson LI, McDermott JL, Kucera J, Walro JM (2002). Striatal dopamine output is compromised within ± BDNF mice. Synapse.

[16] Donovan MJ, Lin MI, Wiegn P, Ringstedt T, Kraemer R, Hahn R, Wang S, Ibanez CF, Rafii S, Hempstead BL (2000). Brain derived neurotrophic factor is an endothelial cell survival factor required for intramyocardial vessel stabilization. Development.

[17] Dunn KC, Aotaki-Keen AE, Putkey FR, Hjelmeland LM (1996). ARPE-19, a human retinal pigment epithelial cell line with differentiated properties. Exp Eye Res.

[18] Ebert MS, Neilson JR, Sharp PA (2007). MicroRNA sponges: competitive inhibitors of small RNAs in mammalian cells. Nat Methods.

[19] Egan MF, Kojima M, Callicott JH, Goldberg TE, Kolachana BS, Bertolino A, Zaitsev E, Gold B, Goldman D, Dean M, Lu B, Weinberger DR (2003). The BDNF val66met polymorphism affects activity-dependent secretion of BDNF and human memory and hippocampal function. Cell.

[20] Ernfors P, Ibanez CF, Ebendal T, Olson L, Persson H (1990). Molecular cloning and neurotrophic activities of a protein with structural similarities to nerve growth factor: developmental and topographical expression in the brain. Proc Natl Acad Sci USA.

[21] Ernfors P, Lee KF, Jaenisch R (1994). Mice lacking brain-derived neurotrophic factor develop with sensory deficits. Nature.

[22] Ernfors P, Wetmore C, Olson L, Persson H (1990). Identification of cells in rat brain and peripheral tissues expressing mRNA for members of the nerve growth factor family. Neuron.

[23] Ferrer I, Goutan E, Marin C, Rey MJ, Ribalta T (2000). Brain-derived neurotrophic factor in Huntington disease. Brain Res.

[24] Filipowicz W, Bhattacharyya SN, Sonenberg N (2008). Mechanisms of post-transcriptional regulation by microRNAs: are the answers in sight?. Nat Rev Genet.

[25] Friedman LM, Dror AA, Mor E, Tenne T, Toren G, Satoh T, Biesemeier DJ, Shomron N, Fekete DM, Hornstein E, Avraham KB (2009). MicroRNAs are essential for development and function of inner ear hair cells in vertebrates. Proc Natl Acad Sci USA.

[26] Fukuchi M, Tsuda M (2010). Involvement of the 3′-untranslated region of the brain-derived neurotrophic factor gene in activity-dependent mRNA stabilization. J Neurochem.

[27] Funakoshi H, Frisen J, Barbany G, Timmusk T, Zachrisson O, Verge VM, Persson H (1993). Differential expression of mRNAs for neurotrophins and their receptors after axotomy of the sciatic nerve. J Cell Biol.

[28] Gentner B, Schira G, Giustacchini A, Amendola M, Brown BD, Ponzoni M, Naldini L (2009). Stable knockdown of microRNA in vivo by lentiviral vectors. Nat Methods.

[29] Grimson A, Farh KK, Johnston WK, Garrett-Engele P, Lim LP, Bartel DP (2007). MicroRNA targeting specificity in mammals: determinants beyond seed pairing. Mol Cell.

[30] He M, Liu Y, Wang X, Zhang MQ, Hannon GJ, Huang ZJ (2012). Cell-type-based analysis of microRNA profiles in the mouse brain. Neuron.

[31] Hofer M, Pagliusi SR, Hohn A, Leibrock J, Barde YA (1990). Regional distribution of brain-derived neurotrophic factor mRNA in the adult mouse brain. EMBO J.

[32] Huang EJ, Reichardt LF (2001). Neurotrophins: roles in neuronal development and function. Annu Rev Neurosci.

[33] John B, Enright AJ, Aravin A, Tuschl T, Sander C, Marks DS (2004). Human MicroRNA targets. PLoS Biol.

[34] Kertesz M, Iovino N, Unnerstall U, Gaul U, Segal E (2007). The role of site accessibility in microRNA target recognition. Nat Genet.

[35] Kim HK, Lee YS, Sivaprasad U, Malhotra A, Dutta A (2006). Muscle-specific microRNA miR-206 promotes muscle differentiation. J Cell Biol.

[36] Konopka W, Kiryk A, Novak M, Herwerth M, Parkitna JR, Wawrzyniak M, Kowarsch A, Michaluk P, Dzwonek J, Arnsperger T, Wilczynski G, Merkenschlager M, Theis FJ, Kohr G, Kaczmarek L, Schutz G (2010). MicroRNA loss enhances learning and memory in mice. J Neurosci.

[37] Korte M, Carroll P, Wolf E, Brem G, Thoenen H, Bonhoeffer T (1995). Hippocampal long-term potentiation is impaired in mice lacking brain-derived neurotrophic factor. Proc Natl Acad Sci USA.

[38] Krek A, Grun D, Poy MN, Wolf R, Rosenberg L, Epstein EJ, MacMenamin P, da Piedade I, Gunsalus KC, Stoffel M, Rajewsky N (2005). Combinatorial microRNA target predictions. Nat Genet.

[39] Lagos-Quintana M, Rauhut R, Yalcin A, Meyer J, Lendeckel W, Tuschl T (2002). Identification of tissue-specific microRNAs from mouse. Curr Biol.

[40] Landgraf P, Rusu M, Sheridan R, Sewer A, Iovino N, Aravin A, Pfeffer S, Rice A, Kamphorst AO, Landthaler M, Lin C, Socci ND, Hermida L, Fulci V, Chiaretti S, Foa R, Schliwka J, Fuchs U, Novosel A, Muller RU, Schermer B, Bissels U, Inman J, Phan Q, Chien M, Weir DB, Choksi R, De Vita G, Frezzetti D, Trompeter HI, Hornung V, Teng G, Hartmann G, Palkovits M, Di Lauro R, Wernet P, Macino G, Rogler CE, Nagle JW, Ju J, Papavasiliou FN, Benzing T, Lichter P, Tam W, Brownstein MJ, Bosio A, Borkhardt A, Russo JJ, Sander C, Zavolan M, Tuschl T (2007). A mammalian microRNA expression atlas based on small RNA library sequencing. Cell.

[41] Lau AG, Irier HA, Gu J, Tian D, Ku L, Liu G, Xia M, Fritsch B, Zheng JQ, Dingledine R, Xu B, Lu B, Feng Y (2010). Distinct 3′UTRs differentially regulate activity-dependent translation of brain-derived neurotrophic factor (BDNF). Proc Natl Acad Sci USA.

[42] Lee ST, Chu K, Jung KH, Kim JH, Huh JY, Yoon H, Park DK, Lim JY, Kim JM, Jeon D, Ryu H, Lee SK, Kim M, Roh JK (2012). miR-206 regulates brain-derived neurotrophic factor in Alzheimer disease model. Ann Neurol.

[43] Lewis BP, Burge CB, Bartel DP (2005). Conserved seed pairing, often flanked by adenosines, indicates that thousands of human genes are microRNA targets. Cell.

[44] Lu B (2003). BDNF and activity-dependent synaptic modulation. Learn Mem.

[45] Lyons WE, Mamounas LA, Ricaurte GA, Coppola V, Reid SW, Bora SH, Wihler C, Koliatsos VE, Tessarollo L (1999). Brain-derived neurotrophic factor-deficient mice develop aggressiveness and hyperphagia in conjunction with brain serotonergic abnormalities. Proc Natl Acad Sci USA.

[46] Mellios N, Huang HS, Grigorenko A, Rogaev E, Akbarian S (2008). A set of differentially expressed miRNAs, including miR-30a-5p, act as post-transcriptional inhibitors of BDNF in prefrontal cortex. Hum Mol Genet.

[47] Miura P, Amirouche A, Clow C, Belanger G, Jasmin BJ (2012). Brain-derived neurotrophic factor expression is repressed during myogenic differentiation by miR-206. J Neurochem.

[48] Moore MJ (2005). From birth to death: the complex lives of eukaryotic mRNAs. Science.

[49] Mousavi K, Jasmin BJ (2006). BDNF is expressed in skeletal muscle satellite cells and inhibits myogenic differentiation. J Neurosci.

[50] Nakajo Y, Miyamoto S, Nakano Y, Xue JH, Hori T, Yanamoto H (2008). Genetic increase in brain-derived neurotrophic factor levels enhances learning and memory. Brain Res.

[51] Park H, Poo MM (2013). Neurotrophin regulation of neural circuit development and function. Nat Rev Neurosci.

[52] Peltier HJ, Latham GJ (2008). Normalization of microRNA expression levels in quantitative RT-PCR assays: identification of suitable reference RNA targets in normal and cancerous human solid tissues. RNA.

[53] Poliseno L, Salmena L, Zhang J, Carver B, Haveman WJ, Pandolfi PP (2010). A coding-independent function of gene and pseudogene mRNAs regulates tumour biology. Nature.

[54] Radzikinas K, Aven L, Jiang Z, Tran T, Paez-Cortez J, Boppidi K, Lu J, Fine A, Ai X (2011). A Shh/miR-206/BDNF cascade coordinates innervation and formation of airway smooth muscle. J Neurosci.

[55] Rios M, Fan G, Fekete C, Kelly J, Bates B, Kuehn R, Lechan RM, Jaenisch R (2001). Conditional deletion of brain-derived neurotrophic factor in the postnatal brain leads to obesity and hyperactivity. Mol Endocrinol.

[56] Saetrom P, Heale BS, Snove O, Aagaard L, Alluin J, Rossi JJ (2007). Distance constraints between microRNA target sites dictate efficacy and cooperativity. Nucleic Acids Res.

[57] Salmena L, Poliseno L, Tay Y, Kats L, Pandolfi PP (2011). A ceRNA hypothesis: the Rosetta Stone of a hidden RNA language?. Cell.

[58] Sandberg R, Neilson JR, Sarma A, Sharp PA, Burge CB (2008). Proliferating cells express mRNAs with shortened 3′ untranslated regions and fewer microRNA target sites. Science.

[59] Sempere LF, Freemantle S, Pitha-Rowe I, Moss E, Dmitrovsky E, Ambros V (2004). Expression profiling of mammalian microRNAs uncovers a subset of brain-expressed microRNAs with possible roles in murine and human neuronal differentiation. Genome Biol.

[60] Siepel A, Bejerano G, Pedersen JS, Hinrichs AS, Hou M, Rosenbloom K, Clawson H, Spieth J, Hillier LW, Richards S, Weinstock GM, Wilson RK, Gibbs RA, Kent WJ, Miller W, Haussler D (2005). Evolutionarily conserved elements in vertebrate, insect, worm, and yeast genomes. Genome Res.

[61] Timmusk T, Palm K, Metsis M, Reintam T, Paalme V, Saarma M, Persson H (1993). Multiple promoters direct tissue-specific expression of the rat BDNF gene. Neuron.

[62] Timmusk T, Persson H, Metsis M (1994). Analysis of transcriptional initiation and translatability of brain-derived neurotrophic factor mRNAs in the rat brain. Neurosci Lett.

[63] Trang T, Beggs S, Salter MW (2011). Brain-derived neurotrophic factor from microglia: a molecular substrate for neuropathic pain. Neuron Glia Biology.

[64] Tyler WJ, Alonso M, Bramham CR, Pozzo-Miller LD (2002). From acquisition to consolidation: on the role of brain-derived neurotrophic factor signaling in hippocampal-dependent learning. Learn Mem.

[65] Yaffe D, Saxel O (1977). Serial passaging and differentiation of myogenic cells isolated from dystrophic mouse muscle. Nature.

[66] Yajima Y, Narita M, Usui A, Kaneko C, Miyatake M, Yamaguchi T, Tamaki H, Wachi H, Seyama Y, Suzuki T (2005). Direct evidence for the involvement of brain-derived neurotrophic factor in the development of a neuropathic pain-like state in mice. J Neurochem.

[67] Zhao Y, Ransom JF, Li A, Vedantham V, von Drehle M, Muth AN, Tsuchihashi T, McManus MT, Schwartz RJ, Srivastava D (2007). Dysregulation of cardiogenesis, cardiac conduction, and cell cycle in mice lacking miRNA-1-2. Cell.

[68] Zhao Y, Samal E, Srivastava D (2005). Serum response factor regulates a muscle-specific microRNA that targets Hand2 during cardiogenesis. Nature.

[69] Zuccato C, Ciammola A, Rigamonti D, Leavitt BR, Goffredo D, Conti L, MacDonald ME, Friedlander RM, Silani V, Hayden MR, Timmusk T, Sipione S, Cattaneo E (2001). Loss of huntingtin-mediated BDNF gene transcription in Huntington’s disease. Science.

