# Supplementary material for: miR-1, miR-10b, miR-155, and miR-191 are novel regulators of BDNF
Source: Cell Mol Life Sci. 2014 May 8;71(22):4443–56. doi: 10.1007/s00018-014-1628-x (PMC4207943; doi:10.1007/s00018-014-1628-x)
Supplement: Supplementary file 1 — Supplementary material 1 (PDF 74 kb) [file 18_2014_1628_MOESM1_ESM.pdf]

miR-1, miR-10b, miR-155 and miR-191 are novel regulators of BDNF

Cellular and Molecular Life Sciences
Kärt Varendi, Anmol Kumar, Mari-Anne Härma and Jaan-Olle Andressoo*
Institute of Biotechnology, University of Helsinki, 00014, Finland
*To whom correspondence should be addressed. Tel: +358 50 358 1213; E-mail: jaan-olle.andressoo@helsinki.fi

ELECTRONIC SUPPLEMENTARY MATERIAL

Online resource 1 List of PCR and qPCR primers used in the study.

Online resource 2 Conservation of miR binding sites and flanking sequences in BDNF 3'UTR. Light blue background: conserved nucleotides within sequence complementary to miR seed; grey background: conservation within 20 nucleotides flanking miR seed sequence.

Online resource 3 **A**. List of broadly conserved microRNA families. **B**. List of miRs from broadly conserved miR families. **C**. Potential miR binding sites within Mus musculus BDNF 3'UTR predicted with different bioinformatics tools. In all analyses, only predictions for miRs from broadly conserved miR families expressed in Mus musculus are shown. In PITA analysis, sites with ddG<-10 and ddG<0 are indicated in columns D and E, respectively. In miRanda analysis only sites of conserved miRs with good mirSVR scores are shown. TargetScan ((Lewis et al. 2005), www.targetscan.org), PITA ((Kertesz et al. 2007), http://genie.weizmann.ac.il/pubs/mir07/index.html), miRanda ((John et al. 2004), www.microrna.org), PicTar ((Krek et al. 2005), http://pictar.mdc-berlin.de/). mmu – *Mus musculus.*

Online resource 4 **(a)** BDNF mRNA expression in different cell lines and mouse hippocampus, relative to β-actin. N=3-5 **(b)** Conservation between miR-1 and miR-206 in mouse (mmu) and human (hsa). miR seed sequence is underlined and conserved nucleotides are shown in bold. **(c)** Overview of the induction of myogenic differentiation and timeline of RNA/protein isolation and luciferase assays. Images of undifferentiated myoblasts and differentiated myotubes of C2C12 mouse skeletal muscle cell line. **(d)** BDNF protein levels in C2C12 myoblasts and myotubes normalized to total protein content. N=3. **(e)** Expression of miR-1 and miR-206 in C2C12 myoblasts and myotubes normalized to sno202. N=2. Error bars denote mean ± SEM; *p<0.05.

Online resource 5 miR expression in different cells, normalized to miR-191 (human cells) or sno-202 (mouse cells). N=3-5. Values are presented as mean ± SEM. n.e – not expressed; n.t – not tested.

Online resource 6 miR binding sites within SV40 late polyA sequence predicted with PITA. Only predictions for miRs from broadly conserved miR families with ddG<-5 are shown. mmu – *Mus musculus.*
