# Supplementary material for: miR-1, miR-10b, miR-155, and miR-191 are novel regulators of BDNF
Source: Cell Mol Life Sci. 2014 May 8;71(22):4443–56. doi: 10.1007/s00018-014-1628-x (PMC4207943; doi:10.1007/s00018-014-1628-x)
Supplement: Supplementary file 2 — Supplementary material 2 (PDF 12 kb) [file 18_2014_1628_MOESM2_ESM.pdf]

**miR-1, miR-10b, miR-155 and miR-191 are novel regulators of BDNF**

Cellular and Molecular Life Sciences

Kärt Varendi, Anmol Kumar, Mari-Anne Härma and Jaan-Olle Andressoo*
Institute of Biotechnology, University of Helsinki, 00014, Finland
*To whom correspondence should be addressed. Tel. +358 50 358 1213; E-mail: jaan-olle.andressoo@helsinki.fi

**Online resource 1**

List of used PCR and qPCR primers

| **PCR primers** | Forward | Reverse |
| --- | --- | --- |
| BDNF 3'UTR | GCTTCTAGATGGATTTATGTTGTATAGATTATATTG | CACTCTAGAACTTTGAAAATATATTTAAAAACATTAA |
| BDNF short 3'UTR | GCATCTAGATGGATTTATGTTGTATAGATTATAT | CAGTCTAGAATCTGTTTTCTGAAAGAGGGAC |
| miR-1-1 mutant | **TCATCCA**TCGATAATGTTGTGGTTTGTTGC | AATGCAGACTTTTAAAGTTGTGC |
| miR-1-2/30/191 mutant | **TCACT**CATTTTAGACACTAAGTATCTTCG | **AACTA**GTTTTGGTTCCAATTTTTGTTTTTG |
| miR-1-3 mutant | **TCATCCA**TAAAGGAAGGCTCGGAAGCAC | CTCAAGTACCATTCCCCACC |
| miR-10 mutant | **TGAGCAAG**AATTATTCAGTTAAGAAAAAATAAT | TATGTATATATACAAATAGATAATT |
| miR-155 mutant | TAACCACATCAGAAGCCTATTG | AGTTTTAAT**AT**CAATTTTTTCAATAAC |

| **QPCR primers** | Forward | Reverse | Accession number | Amplicon length | Efficiency |
|---|---|---|---|---|---|
| Homo sapiens actin, beta | CCAACCGCGAGAAGATGA | CCAGAGGCGTACAGGGATAG | NM_001101.3 | 97 nt | 1,942 |
| Homo sapiens BDNF (long 3'UTR, transcript variants 1-18) | GAGCCCTGTATCAACCCAGAA | TGCCAACTCCACATAGCCTC | NM_170735.5 | 100 nt | 1,902 |
| Homo sapiens BDNF (CDS, transcript variants 1-18) | GTAACGGCGGCAGACAAA | GACCTTTTCAAGGACTGTGACC | NM_170735.5 | 69 nt | 1,798 |
| Mus musculus actin, beta | CCAGTTCGCCATGGATGAC | GAGCCGTTGTCGACGACC | NM_007393.3 | 51 nt | 1,804 |
| Mus musculus BDNF (CDS, transcript variants 1-4) | TACCTGGATGCCGCAAACAT | GCTGTGACCCACTCGCTAAT | NM_007540.4 | 104 nt | 1,919 |
