# Supplementary material for: miR-1, miR-10b, miR-155, and miR-191 are novel regulators of BDNF
Source: Cell Mol Life Sci. 2014 May 8;71(22):4443–56. doi: 10.1007/s00018-014-1628-x (PMC4207943; doi:10.1007/s00018-014-1628-x)
Supplement: Supplementary file 3 — Supplementary material 3 (PDF 568 kb) [file 18_2014_1628_MOESM3_ESM.pdf]

miR-1, miR-10b, miR-155 and miR-191 are novel regulators of BDNF
Cellular and Molecular Life Sciences
Kärt Varendi, Anmol Kumar, Mari-Anne Härma and Jaan-Olle Andressoo*
Institute of Biotechnology, University of Helsinki, 00014, Finland
*To whom correspondence should be addressed. Tel: +358 50 358 1213; E-mail: jaan-olle.andressoo@helsinki.fi

Online resource 2

Alignment of conserved miR target sites within BDNF 3'UTR spanning 20 nt upstream and 20 nt downstream of the miR seed sequence

miR-10ab (*Mus musculus* BDNF 3'UTR nt pos 59-66)

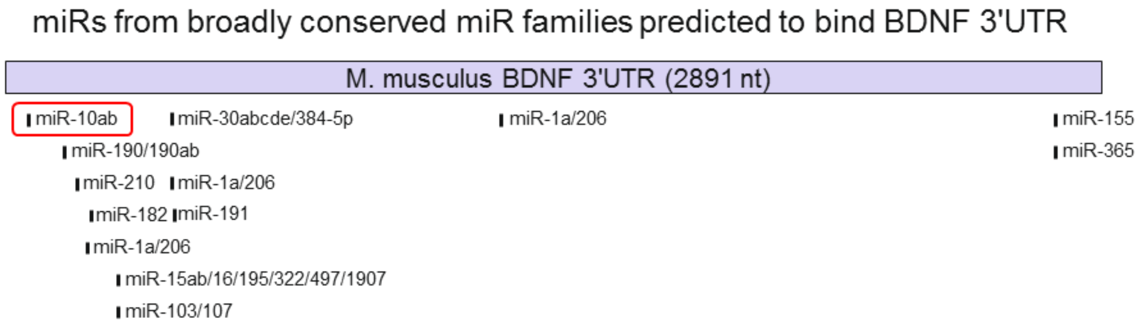

M. musculus (mouse)	U-CUAUUUGUAUAUAUACAUAACAGGGUAAAUUAUUCAGUUAAGAAAAA
H. sapiens (human)	U-CUAUUUGUAUAUAUACAUAACAGGGUAAAUUAUUCAGUUAAGAAAAA
P. troglodytes (chimpanzee)	U-CUAUUUGUAUAUAUACAUAACAGGGUAAAUUAUUCAGUUAAGAAAAA
M. mulatta (rhesus macaque)	U-CUAUUUGUAUAUAUACAUAACAGGGUAAAUUAUUCAGUUAAGAAAAA
O. garnettii (bushbaby)	U-CUAUUUGUAUAUAUACAUAACAGGGUAAAUUAUUCAGUUAAGAAAAA
T. belangeri (treeshrew)	N-CUAUUUGUAUAUAUACAUAACAGGGUAAAUUAUUCAGUUAAGAAAAA
R. norvegicus (rat)	U-CUAUUUGUAUAUAUACAUAACAGGGUAAAUUAUUCAGUUAAGAAAAA
C. porcellus (guinea pig)	U-CUAUUUGUAUAUAUACAUAACAGGGUAAAUUAUUCAGUUAAGAAAAA
O. cuniculus (rabbit)	UUCUAUUUGUAUAUAUACAUAACAGGGUAAAUUAUUCAGUUAAGAAAAA
S. araneus (shrew)	U-CUAUUUGUAUAUAUACAUAACAGGGUAAAUUAUUCAGUUAAGAAAAA
E. europaeus (hedgehog)	U-CUAUUUGUAUAUACACAUAACAGGGUAAAUUAUUCAGUUAAGAAAAA
C. l. familiaris (dog)	U-CUAUUUGUAUAUAUACAUAACAGGGUAAAUUAUUCAGUUAAGAAAAA
F. catus (cat)	U-CUAUUUGUAUAUAUACAUAACAGGGUAAAUUAUUCAGUUAAGAAAAA
E. f. caballus (horse)	U-CUAUUUGUAUAUAUACAUAACAGGGUAAAUUAUUCAGUUAAGAAAAA
B. taurus (cow)	U-CUAUUUGUAUAUAUACAUAACAGGGUAAAUUAUUCAGUUAAGAAAAA
D. rerio (zebrafish)	U-CUAUUUGUAUAUAUACAUAACAGGGUAAAUUAUUCAGUUAAGAAAAA
L. africana (elephant)	U-CUAUUUGUAUAUAUACAUAACAGGGUAAAUUAUUCAGUUAAGAAAAA
E. telfairi (tenrec)	U-CUAUUUGUAUAUAUACUUAACAGGGUAAAUUAUUCAGUUAAGAAAAA
M. domestica (opossum)	U-CUAUUUGUAUAUAUACAUAACAGGGUAAAUUAUUCAGU-AAGAAAAA
O. anatinus (platypus)	U-CUAUUUGUAUAUAUACAUUACAGGGUAAAUUAUUCAGU-AAGAAAAA
A. carolinensis (lizard)	U-CUAUUUGUAUAUAUACAUAACAGGGUAAAUUAUUCAGUAAGAAAGAA
X. tropicalis (frog)	U-CUAUUUGUAUAUAUACAUAACAGGGUAAAUUAUUCCGUAAGAAAAG

miR-190/190ab (*Mus musculus* BDNF 3’UTR nt pos 155-161)

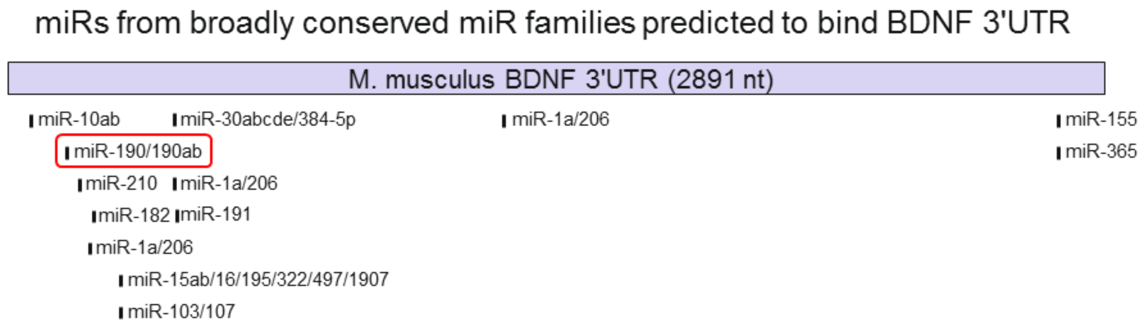


```
M. musculus (mouse)             UUCUACAAUCUAUUUAUUGGACAUAUCCAUG-AC-CUGAAAGG-AAA-------------------CAG
H. sapiens (human)              UUCUACAAUCUAUUUAUUGGACAUGUCCAUG-AC-CAGAAGGG-AAA-------------------CAG
P. troglodytes (chimpanzee)     UUCUACAAUCUAUUUAUUGGACAUGUCCAUG-AC-CAGAAGGG-AAA-------------------CAG
M. mulatta (rhesus macaque)     UUCUACAAUCUAUUUAUUGGACAUGUCCAUG-AC-CAGAAGGG-AAA-------------------CAG
O. garnettii (bushbaby)         UUCUACAAUCUAUUUAUUGGACAUAUCCAUG-AC-CAGAAGGG-AAA-------------------CAG
T. belangeri (treeshrew)        UUCUACAAUCUAUUUAUUGGACAUAUCCAUG-AC-CAGAAGGG-AAA-------------------CAG
R. norvegicus (rat)             UUCUACAAUCUAUUUAUUGGACAUAUCCAUG-AC-CAGAAAG--AAA-------------------CAG
C. porcellus (guinea pig)       UUCUACAACCUAUUUAUUGGACAUAUCCAUG-AC-CAGAAGGAAAAA-------------------CAG
O. cuniculus (rabbit)           UUCUACAAUCUAUUUAUUGGACAUAUCCAUG-AC-CAGAAGGG-AAA-------------------CAG
S. araneus (shrew)              UUCUACAAUCUAUUUAUUGGACAUAUCCAUG-AC-CAGAAGGG-AAA-------------------CAG
E. europaeus (hedgehog)         UUCUACAAUCUAUUUAUUGGACACAUCCAUG-AC-CAGAAGGG-AAA-------------------CAG
C. l. familiaris (dog)          UUCUACAAUCUAUUUAUUGGACAUAUCCAUG-AC-CAGAAGGG-AAA-------------------CAG
F. catus (cat)                  UUCUACAAUCUAUUUAUUGGACAUAUCCAUG-AC-CAGAAGGG-AAA-------------------CAG
E. f. caballus (horse)          UUCUACAGUCUGUUUAUUGGACAGAUCCAUG-AC-CAGAAGGG-AAA-------------------CAG
B. taurus (cow)                 UUCUACAAUCUAUUUAUUGGACAUUUCCAUG-AC-CAGAAGGG-AAA-------------------CAG
D. rerio (zebrafish)            UUCUACAAUCUAUUUAUUGGACAUAUCCAUGUACACAGAAGGA-AAA-------------------CAG
L. africana (elephant)          UUCUACAAUCUAUUUAUUGGACAUAUCCAUG-AC-CAGAAGGG-AAA-------------------CAG
E. telfairi (tenrec)            UUCUACACUCUAUUUAUUGGACAUAUCCCUG-AC-CAGAAGGG-AAA-------------------CAG
M. domestica (opossum)          UUCUACAAUCUAUUUAUUAAACAUAUCCAUG-AC-CUGAAGGG-AAA-------------------CAG
O. anatinus (platypus)          UUCUACAAUCUAUUUAUUGAACAUAUCCAUG-AC-CUGAAGGG-AAA-------------------UAG
A. carolinensis (lizard)        UUCCACAAUCUAUUUAUUGAACAUAUCCAUG-AC-CUAGAGGG-GAACAAAAGGAGGAAAAAAAGAUAU
X. tropicalis (frog)            UUCUACAAUCUAUUUAUUGAACAUAUCCAUA-AC--AUAAAGA-AAC-------------------CAG
```

miR-210 (*Mus musculus* BDNF 3’UTR nt pos 189-195)

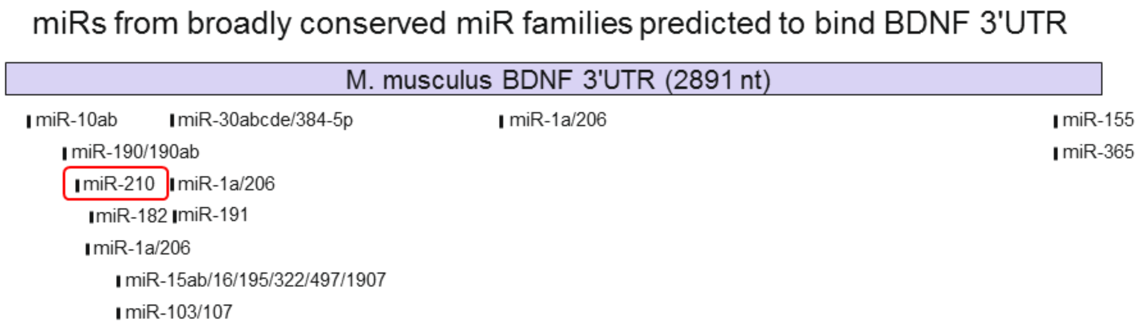


```
M. musculus (mouse)              UGAAAGG-AAA-------------------CAG--UCAUU---U-GCGCACAACUUU-----AAAA---------G--------------------UCUGCAUUACA
H. sapiens (human)               AGAAGGG-AAA-------------------CAG--UCAUU---U-GCGCACAACUU------AAAA---------A---------------G----UCUGCAUUACA
P. troglodytes (chimpanzee)      AGAAGGG-AAA-------------------CAG--UCAUU---U-GCGCACAACUU------AAAA---------A---------------G----UCUGCAUUACA
M. mulatta (rhesus macaque)      AGAAGGG-AAA-------------------CAG--UCAUU---U-GCGCACAACUUU-----AAAA---------A---------------G----UCUGCAUUACA
O. garnettii (bushbaby)          AGAAGGG-AAA-------------------CAG--UCAUU---U-GCGCACAACUUUGAAAAAAAA---------A---------------G----UUUGCAUUACA
T. belangeri (treeshrew)         AGAAGGG-AAA-------------------CAG--UCAUU---U-GCGCACAACUUU-----ACAA---------A---------------G----UCUGCAUUACA
R. norvegicus (rat)              AGAAAG--AAA-------------------CAG--UCAUU---U-GCGCACAACUUU-----AAAA---------G--------------------UCUGCAUUACA
C. porcellus (guinea pig)        AGAAGGAAAAA-------------------CAG--UCAUU---U-GCGCACAACCUU-----AAAC---------A---------------AAAAGUCUGCAUUACA
O. cuniculus (rabbit)            AGAAGGG-AAA-------------------CAG--UCAUU---U-GCGCACAACUUU-----AAAA---------U---------------G----UCUGCAUUACA
S. araneus (shrew)               AGAAGGG-AAA-------------------CAG--UCAUU--UU-GCGCACAACUUA-----AAAA---------AAAAA---------AAG----UCUGCAUUACA
E. europaeus (hedgehog)          AGAAGGG-AAA-------------------CAG--UCAUU--UUUGCGCACAACUUU-----AAAA---------A---------------G----UCUGCAUUACA
C. l. familiaris (dog)           AGAAGGG-AAA-------------------CAG--UCAUU--UU-GCGCACAACUUU-----AAAA---------A---------------G----UCUGCAUUACA
F. catus (cat)                   AGAAGGG-AAA-------------------CAG--UCAUU--UU-GCGCACAACUUU-----AAAA---------A---------------G----UCUGCAUUUCA
E. f. caballus (horse)           AGAAGGG-AAA-------------------CAG--UCAUU--UC-GCGCACAACUUU-----AAAA---------A---------------A----UCUGCAUUACA
B. taurus (cow)                  AGAAGGG-AAA-------------------CAG--UCAUUUUUU-GCGCACAACUUU-----AAAA---------A---------------G----UCUGCUUUACA
D. rerio (zebrafish)             AGAAGGA-AAA-------------------CAG--UCAUU---U-GCGCACAACUUU-----AAAA---------A---------------G----UCUGCAUUACA
L. africana (elephant)           AGAAGGG-AAA-------------------CAG--UCAUU---U-GCGCACAACUUU------AAA---------A---------------G----UCUGCAUUACA
E. telfairi (tenrec)             AGAAGGG-AAA-------------------CAG--UCAUU---C-GCGCACAACUU-------AAA---------A---------------G----UCUGCAUUACA
M. domestica (opossum)           UGAAGGG-AAA-------------------CAGAGUCAUU---U-GCGCACAACUGA-----AAAA---------AAAAA--------UAAG----UCUGCAUUACA
O. anatinus (platypus)           UGAAGGG-AAA-------------------UAG--UCAUU---U-GCGCACAACUUA-----AAAA---------AAAAACAACAAAAUAAG----UCUGCAUUACA
A. carolinensis (lizard)         UAGAGGG-GAACAAAAGGAGGAAAAAAAGAUAU--CCAUU---U-GCGCACAACUUU-----GAGAAACAAAACAA---------------A----UUUGCAUUACA
X. tropicalis (frog)             AUAAAGA-AAC-------------------CAGAGUCAUC---U-GUGCACAAUGUA-----A-AA---------A---------------G----CCUGCGUUAUG
```

miR-1a/206, site 1 (*Mus musculus* BDNF 3’UTR nt pos 213-219)

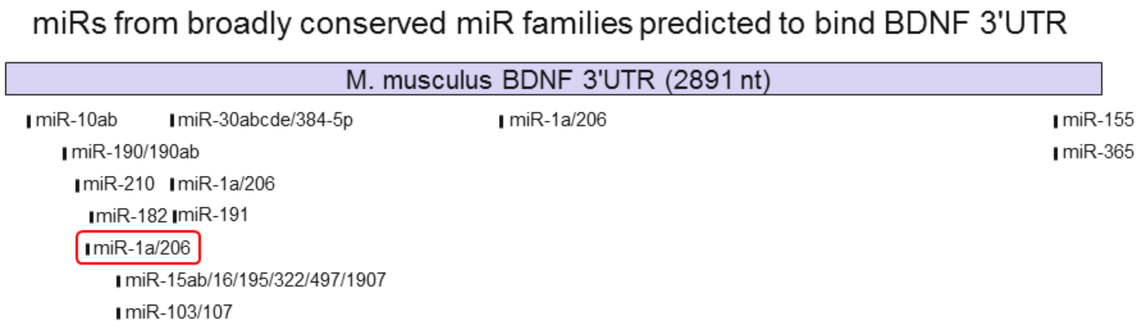


```
M. musculus (mouse)              CAACUUU-----AAAA---------G-------------------UCUGCAUUACAUUCCUCGAUAAU--GUUGUGGUUUGU
H. sapiens (human)               CAACUU------AAAA---------A---------------G----UCUGCAUUACAUUCCUUGAUAAU--GUUGUGGUUUGU
P. troglodytes (chimpanzee)      CAACUU------AAAA---------A---------------G----UCUGCAUUACAUUCCUUGAUAAU--GUUGUGGUUUGU
M. mulatta (rhesus macaque)      CAACUUU-----AAAA---------A---------------G----UCUGCAUUACAUUCCUUGAUAAU--GUUGUGGUUUGU
O. garnettii (bushbaby)          CAACUUUGAAAAAAAA---------A---------------G----UUUGCAUUACAUUCCUCGAUAAU--GUUGUGGUUUGU
T. belangeri (treeshrew)         CAACUUU-----ACAA---------A---------------G----UCUGCAUUACAUUCCUCGAUAAU--GUUGUGGUUUGU
R. norvegicus (rat)              CAACUUU-----AAAA---------G-------------------UCUGCAUUACAUUCCUCGAUAAU--GUUGUGGUUUGU
C. porcellus (guinea pig)        CAACCUU-----AAAC---------A---------------AAAAGUCUGCAUUACAUUCCUCGAUCAU--GUUGUGGUUUGU
O. cuniculus (rabbit)            CAACUUU-----AAAA---------U---------------G----UCUGCAUUACAUUCCUCAAUAAC--GUUGUGGUUUGU
S. araneus (shrew)               CAACUUA-----AAAA---------AAAAA---------AAG----UCUGCAUUACAUUCCUCGAUAAU--GUUGUGGUUUGU
E. europaeus (hedgehog)          CAACUUU-----AAAA---------A---------------G----UCUGCAUUACAUUCCUUAAUAAU--GU---GGUUUGU
C. l. familiaris (dog)           CAACUUU-----AAAA---------A---------------G----UCUGCAUUACAUUCCUCGAUAAUAUGUUGUGGUUUGU
F. catus (cat)                   CAACUUU-----AAAA---------A---------------G----UCUGCAUUUCAUUCCUCGAUAAU--GUUGUGGUUUGU
E. f. caballus (horse)           CAACUUU-----AAAA---------A---------------A----UCUGCAUUACAUUCCUCGAUAAU--GUUGUGGUUUGU
B. taurus (cow)                  CAACUUU-----AAAA---------A---------------G----UCUGCUUUACAUUCCUC---AAU--GUUGUGGUUUGU
D. rerio (zebrafish)             CAACUUU-----AAAA---------A---------------G----UCUGCAUUACAU-------------------------
L. africana (elephant)           CAACUUU------AAA---------A---------------G----UCUGCAUUACAUUCCUCGAUCAU--GUUGUGGUUUGU
E. telfairi (tenrec)             CAACUU-------AAA---------A---------------G----UCUGCAUUACAUUCCUCGAUAAC--GUUGUGGUUUGU
M. domestica (opossum)           CAACUGA-----AAAA---------AAAAA--------UAAG----UCUGCAUUACAUUCCUCGAUAAC--AUUGUGGUUUGU
O. anatinus (platypus)           CAACUUA-----AAAA---------AAAAACAACAAAAUAAG----UCUGCAUUACAUUCCUCGAUAAC--AUUGUGGUUUGU
A. carolinensis (lizard)         CAACUUU-----GAGAAACAAAACAA---------------A----UUUGCAUUACAUUCCUC--UAAC--AUUGUGGUUUGU
X. tropicalis (frog)             CAAUGUA-----A-AA---------A---------------G----CCUGCGUUAUGUUCCUCAAUAAC--AUUGUGGUUUGU
```

miR-182 (*Mus musculus* BDNF 3'UTR nt pos 245-251)

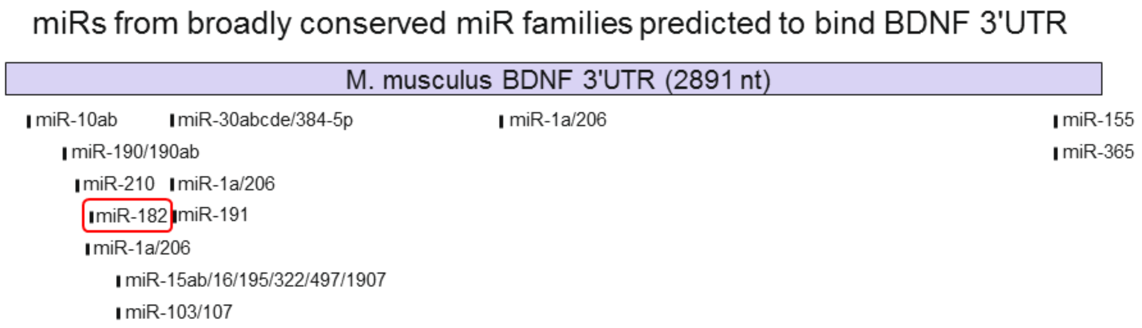


```
M. musculus (mouse)            AAU--GUUGUGGUUUGUUGCCGUUGCCAAGAAUUGAAAA-C--------AAAAA----------GUUU
H. sapiens (human)             AAU--GUUGUGGUUUGUUGCCGUUGCCAAGAACUGAAAA-CAU------AAAAA----------GUU-
P. troglodytes (chimpanzee)    AAU--GUUGUGGUUUGUUGCCGUUGCCAAGAAUUGAAAA-CAU------AAAAA----------GUU-
M. mulatta (rhesus macaque)    AAU--GUUGUGGUUUGUUGCCGUUGCCAAGAAUUGAAAA-CAU------AAAAA----------GUUU
O. garnettii (bushbaby)        AAU--GUUGUGGUUUGUUGCCGUUGCCAAGAAUUGAAA--CAU------AAAAA----------GUUA
T. belangeri (treeshrew)       AAU--GUUGUGGUUUGUUGCCGUUGCCAAGAAUUGAAAA-CAU------AAAAA----------GUUU
R. norvegicus (rat)            AAU--GUUGUGGUUUGUUGCCGUUGCCAAGAAUUGAAAA-C--------AAAAA----------GUUA
C. porcellus (guinea pig)      CAU--GUUGUGGUUUGUUGCCGUUGCCAAGAAUCGAAAA-CAU------AAAAA----------GUUU
O. cuniculus (rabbit)          AAC--GUUGUGGUUUGUUGCCAUUGCCAAGAAUUGAAAA-CAU------AAAAA----------GUU-
S. araneus (shrew)             AAU--GUUGUGGUUUGUUGCCGUUGCCAAGAAUUGAAAA-CGU------AAAAA----------GUUU
E. europaeus (hedgehog)        AAU--GU---GGUUUGUUGCCGUUGCCAAGAAUUGAAAA-CGU------AAAAA----------GUUU
C. l. familiaris (dog)         AAUAUGUUGUGGUUUGUUGCCGUUGCCAAGAAUUGAAAA-CGU------AAAAA----------GUCU
F. catus (cat)                 AAU--GUUGUGGUUUGUUGCCGUUGCCAAGAAUUGAAAA-CGU------AAAAA----------GUUU
E. f. caballus (horse)         AAU--GUUGUGGUUUGUUGCCGUUGCCAAGAAUUGAAAA-CGU------AAAAA----------GUUU
B. taurus (cow)                AAU--GUUGUGGUUUGUUGCCGUUGCCAAGAAUUGAAAAACGU------AAAAAAAAAAAAAAAGUUU
D. rerio (zebrafish)           --------------------------------------------------------------------
L. africana (elephant)         CAU--GUUGUGGUUUGUUGCCGUUGCCAAGAAAUGAAAA-CGU------AAAAA----------GUUA
E. telfairi (tenrec)           AAC--GUUGUGGUUUGUUGCCGUUGCCAAGAAUUGAAAA-CGU------AAAAA----------GUUU
M. domestica (opossum)         AAC--AUUGUGGUUUGUUGCCGUUGCCAAGAAUUGAAAA-CGUUAAAAAAAAAA----------GUUG
O. anatinus (platypus)         AAC--AUUGUGGUUUGUUGCCGUUGCCAAGAAUUGAAAA-CGU------AAAAA----------GUUG
A. carolinensis (lizard)       AAC--AUUGUGGUUUGUUGCCAUUGCCAAGAAUUGAAAA-CGA------UAAAA--------------
X. tropicalis (frog)           AAC--AUUGUGGUUUGUUGCUGUUGCCAAGAAUUGAAAAGCAU------AAAAA--------------
```

miR-103/107 (*Mus musculus* BDNF 3’UTR nt pos 294-300)

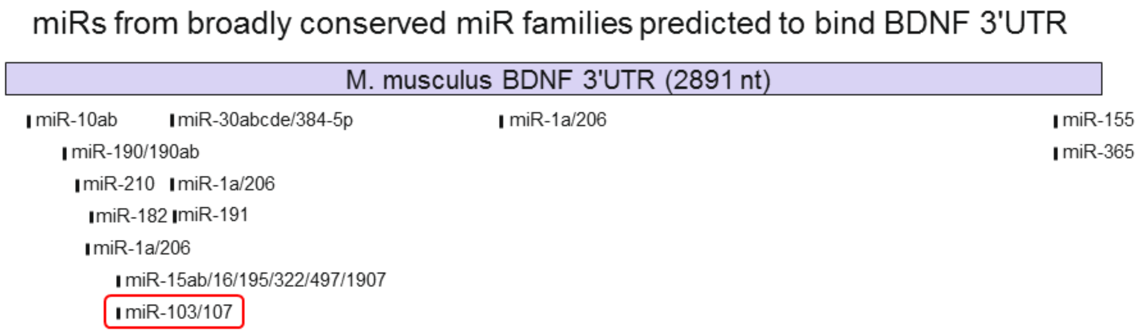


```
M. musculus (mouse)           AAAAA--------------AUAAU------AAU-AA---AUUG-CAUGCUGCUUUAAUUGUGAAUUGAU-AA-U
H. sapiens (human)            AAAAA-----------------AU------AAU-AA---AUUG-CAUGCUGCUUUAAUUGUGAAUUGAU-AA-U
P. troglodytes (chimpanzee)   AAAAA-----------------AU------AAU-AA---AUUG-CAUGCUGCUUUAAUUGUGAAUUGAU-AA-U
M. mulatta (rhesus macaque)   AAAAA-----------------AU------AAU-AA---AUUG-CAUGCUGCUUUAAUUGUGAAUUGAU-AA-U
O. garnettii (bushbaby)       AAAAA-----------------AAAAUAAGAAU-AA---AUUGGCAUGCUGCUUUAAUUGUGA-UUGAU-A--U
T. belangeri (treeshrew)      AAAUA-----------------AU------AAU-AA---AUUG-CAUGCUGCUUUAAUUGUGAAUUGAU-AAAU
R. norvegicus (rat)           AAAAA------------------U------AAU-AA---AUUG-CAUGCUGCUUUAAUUGUGAAUUGAU-AA-U
C. porcellus (guinea pig)     AAAAA--------------UUACU------AAU-AA---AUUG-CAUGCUGCUUUAAUUGUGAAUUGAU-AA-U
O. cuniculus (rabbit)         AAAAA-----------------AU------AAU-AA---AUUG-CAUGCUGCUUUAAUUGUGAAUUGAU-AA-U
S. araneus (shrew)            AAAAU-------------AAUAAU------AAU-AAUAAAUUG-CAUGCUGCUUUAAUUGUGAAUUGAU-AA-U
E. europaeus (hedgehog)       AAACU-------------AAUAAU------AA-------AUUG-CAUGCUGCUUUAAUUGUGAAUUGAU-AA-U
C. l. familiaris (dog)        AAAAAUAAUAAUAAUAAUGAUAAU------AAU-AA---AUUG-CAUGCUGCUUUAAUUGUGAAUCGAU-AA-U
F. catus (cat)                AAAAA-------------AAUAAU------AAU-AA---AUUG-CAUGCUGCUUUAAUUGUGAAUUGAU-AA-U
E. f. caballus (horse)        AA----------------AAUAAU------AAU-AA---AUUG-CAUGCUGCUUUAAUUGUGAAUUGAU-AA-U
B. taurus (cow)               AAAAU-------------ACUACU------AAU-AA---AUUG-CAUGCUGCUUUAAUUGUGAAUUGAU-AA-C
D. rerio (zebrafish)          --------------------------------------------------------------------------
L. africana (elephant)        AAAAA------------UAAUAAU------AAU-AA---AUUG-CAUGCUGCUUUAAUUGUGAAUUGAU-AA-U
E. telfairi (tenrec)          AAAA---------------AUAAU------AAU-AA---AUUG-CAUGCUACUUUAAUGGUGAAUUGAU-AA-U
M. domestica (opossum)        AAAAA-----------------UA------AAU-AA---AUUG-CAUGCUGCUUUAAUUGUGAAUUGAU-AA-U
O. anatinus (platypus)        AAAAA-------------------------AAU-AA---AUUG-CAUGCUGCUUCAAUUGUGAAUUGAUGAA-U
A. carolinensis (lizard)      UUAAA-----------------AA------AAUAAA---AUUG-CAUGCUGCUUAAAUUGUGAAUUGAU-GA-U
X. tropicalis (frog)          UAAAA-----------------AC------CAA-GA---AUUG-CAUGCUGCUUCAGUUGUGAAUUGAC-AA-U
```

miR-15ab/16/195/322/497/1907 (*Mus musculus* BDNF 3’UTR nt pos 295-301)

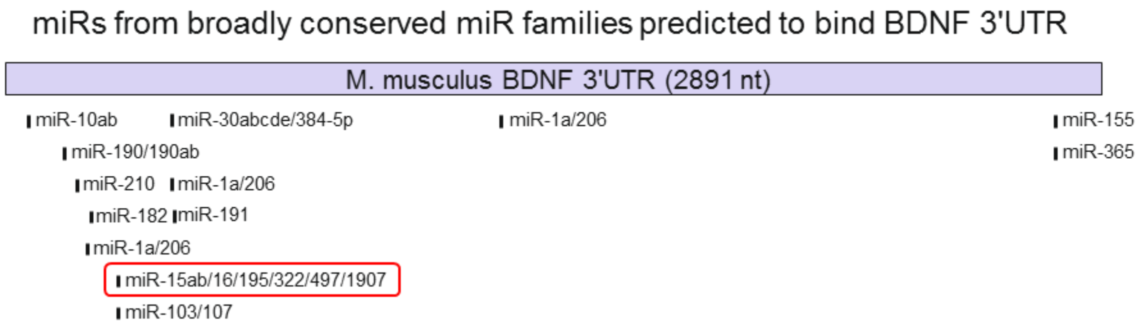


```
M. musculus (mouse)              AAAA--------------AUAAU------AAU-AA---AUUG-CAUGCUGCUUUAAUUGUGAAUUGAU-AA-UA
H. sapiens (human)               AAAA-----------------AU------AAU-AA---AUUG-CAUGCUGCUUUAAUUGUGAAUUGAU-AA-UA
P. troglodytes (chimpanzee)      AAAA-----------------AU------AAU-AA---AUUG-CAUGCUGCUUUAAUUGUGAAUUGAU-AA-UA
M. mulatta (rhesus macaque)      AAAA-----------------AU------AAU-AA---AUUG-CAUGCUGCUUUAAUUGUGAAUUGAU-AA-UA
O. garnettii (bushbaby)          AAAA-----------------AAAAUAAGAAU-AA---AUUGGCAUGCUGCUUUAAUUGUGA-UUGAU-A--UA
T. belangeri (treeshrew)         AAUA-----------------AU------AAU-AA---AUUG-CAUGCUGCUUUAAUUGUGAAUUGAU-AAAUA
R. norvegicus (rat)              AAAA------------------U------AAU-AA---AUUG-CAUGCUGCUUUAAUUGUGAAUUGAU-AA-UA
C. porcellus (guinea pig)        AAAA--------------UUACU------AAU-AA---AUUG-CAUGCUGCUUUAAUUGUGAAUUGAU-AA-UA
O. cuniculus (rabbit)            AAAA-----------------AU------AAU-AA---AUUG-CAUGCUGCUUUAAUUGUGAAUUGAU-AA-UA
S. araneus (shrew)               AAAU-------------AAUAAU------AAU-AAUAAAUUG-CAUGCUGCUUUAAUUGUGAAUUGAU-AA-UA
E. europaeus (hedgehog)          AACU-------------AAUAAU------AA-------AUUG-CAUGCUGCUUUAAUUGUGAAUUGAU-AA-UA
C. l. familiaris (dog)           AAAAUAAUAAUAAUAAUGAUAAU------AAU-AA---AUUG-CAUGCUGCUUUAAUUGUGAAUCGAU-AA-UA
F. catus (cat)                   AAAA-------------AAUAAU------AAU-AA---AUUG-CAUGCUGCUUUAAUUGUGAAUUGAU-AA-UA
E. f. caballus (horse)           A----------------AAUAAU------AAU-AA---AUUG-CAUGCUGCUUUAAUUGUGAAUUGAU-AA-UA
B. taurus (cow)                  AAAU-------------ACUACU------AAU-AA---AUUG-CAUGCUGCUUUAAUUGUGAAUUGAU-AA-CA
D. rerio (zebrafish)             --------------------------------------------------------------------------
L. africana (elephant)           AAAA------------UAAUAAU------AAU-AA---AUUG-CAUGCUGCUUUAAUUGUGAAUUGAU-AA-UA
E. telfairi (tenrec)             AAA---------------AUAAU------AAU-AA---AUUG-CAUGCUACUUUAAUGGUGAAUUGAU-AA-UA
M. domestica (opossum)           AAAA-----------------UA------AAU-AA---AUUG-CAUGCUGCUUUAAUUGUGAAUUGAU-AA-UA
O. anatinus (platypus)           AAAA-------------------------AAU-AA---AUUG-CAUGCUGCUUCAAUUGUGAAUUGAUGAA-UA
A. carolinensis (lizard)         UAAA-----------------AA------AAUAAA---AUUG-CAUGCUGCUUAAAUUGUGAAUUGAU-GA-UA
X. tropicalis (frog)             AAAA-----------------AC------CAA-GA---AUUG-CAUGCUGCUUCAGUUGUGAAUUGAC-AA-UA
```

miR-1a/206, site 2 (*Mus musculus* BDNF 3’UTR nt pos 407-413)

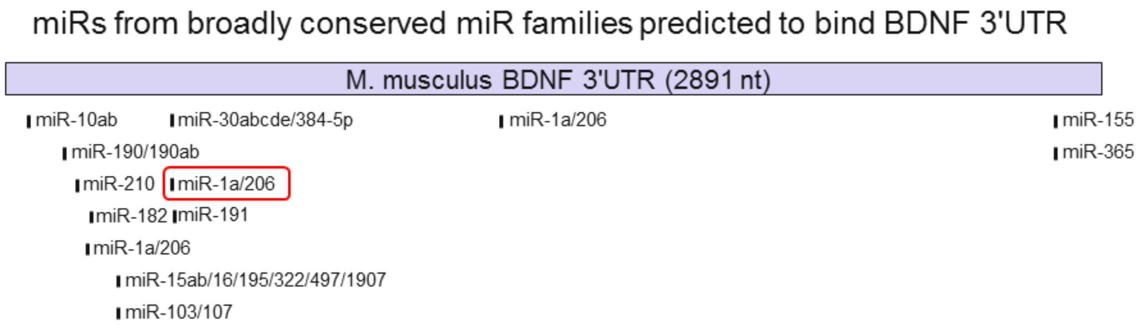


```
M. musculus (mouse)              AAAAAAAAAACAAAAAACAAAAACAAAAAUUGGAACCAAAACAUUCCGUUUACAUUUUAGACACU-AA
H. sapiens (human)               CAACAA--------------------AAAUUUGAACCAAAACAUUCCGUUUACAUUUUAGACAGU-AA
P. troglodytes (chimpanzee)      CAACAA--------------------AAAUUUGAACCAAAACAUUCCGUUUACAUUUUAGACAGU-AA
M. mulatta (rhesus macaque)      CAACAA--------------------AAAUUUGAACCAAAACAUUCCGUUUACAUUUUAGACAGU-AA
O. garnettii (bushbaby)          CAACAA--------------------AAAUUUGAACCCAAACAUUCCGUUUACAUUUAAGACAGU-AA
T. belangeri (treeshrew)         CAACAA--------------------AAAUUUGAACCAAAACAUUCCGUUUCCAUUUUAGACAGU-AC
R. norvegicus (rat)              AAAAAG-----------CAAAAACAAAAAUUUGAACCAAAACAUUCCGUUUACAUUUUAGACACU-AA
C. porcellus (guinea pig)        CACAAA---------------------AAUUUGAACCAAAACAUUCCGUUUACAUUUUAGACAGU-AA
O. cuniculus (rabbit)            ---CAA--------------------AAAUUUGAACCAAAACAUUCCGUUUACAUUUUAGACAGU-AA
S. araneus (shrew)               CAACAA--------------------AAAUUUGAACCAAAACAUUCCGUUUACAUUUUAGACAGU-AA
E. europaeus (hedgehog)          CAACAA--------------------AAAUUUGAACCAAAACAUUCCGUUUACAUUUUAGACAGU-AA
C. l. familiaris (dog)           CGACAA--------------------AAAUUUGAACCAAAACAUUCCGUUUACAUUUUAGACAGU-AA
F. catus (cat)                   CAACAA--------------------AAAUUUGAACCAAAACAUUCCGUUUACAUUUUAGACAGG-AA
E. f. caballus (horse)           CAACAA--------------------AAAUUUGAACCAAAACAUUCCGUUUACAUUUUAGACAGU-AA
B. taurus (cow)                  UAACAA--------------------AAAUUUGAACCAAAACAUUCCGUUUACAUUUUAGACAGU-AA
D. rerio (zebrafish)             --------------------------------------------------------------------
L. africana (elephant)           CAACAA--------------------AAAUUUGAACCAAAACAUCCCGUUUACAUUUUAGACAGU-AA
E. telfairi (tenrec)             CAACAA--------------------AAAUUUGAACCAAAACAUUCCGUUUACAUUUUAGACAGU-AA
M. domestica (opossum)           CACAAC--------------------AAAUUUGAACCCAAACAUUCCGUUUCCAUUUUAGACAGUAAA
O. anatinus (platypus)           AA------------------------AAAUUUGAACC-AAACAUUCCGUUUACAUUUUAGACAGU-AA
A. carolinensis (lizard)         -A-------------------------AAAUUGAACCAAAACAUUCCGUUUACAUUUUAGACAGU-AA
X. tropicalis (frog)             -A------------------------CAAUUUGAACCAAAACAUUCCGUUUACAUUUCACACAGU-A-
```

miR-191 (*Mus musculus* BDNF 3’UTR nt pos 410-416)

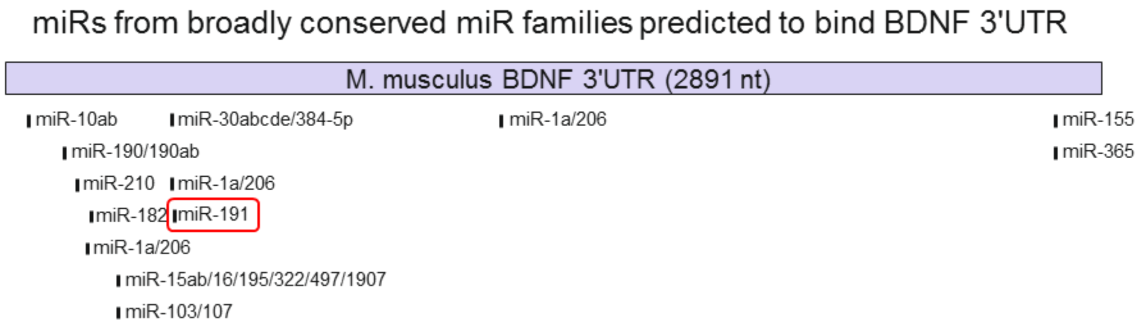


```
M. musculus (mouse)              AAAAAAACAAAAAACAAAAACAAAAAUUGGAACCAAAACAUUCCGUUUACAUUUUAGACACU-AAGUA
H. sapiens (human)               CAA--------------------AAAUUUGAACCAAAACAUUCCGUUUACAUUUUAGACAGU-AAGUA
P. troglodytes (chimpanzee)      CAA--------------------AAAUUUGAACCAAAACAUUCCGUUUACAUUUUAGACAGU-AAGUA
M. mulatta (rhesus macaque)      CAA--------------------AAAUUUGAACCAAAACAUUCCGUUUACAUUUUAGACAGU-AAGUA
O. garnettii (bushbaby)          CAA--------------------AAAUUUGAACCCAAACAUUCCGUUUACAUUUAAGACAGU-AAGAU
T. belangeri (treeshrew)         CAA--------------------AAAUUUGAACCAAAACAUUCCGUUUCCAUUUUAGACAGU-ACGUA
R. norvegicus (rat)              AAG-----------CAAAAACAAAAAUUUGAACCAAAACAUUCCGUUUACAUUUUAGACACU-AAGUA
C. porcellus (guinea pig)        AAA---------------------AAUUUGAACCAAAACAUUCCGUUUACAUUUUAGACAGU-AAGUA
O. cuniculus (rabbit)            CAA--------------------AAAUUUGAACCAAAACAUUCCGUUUACAUUUUAGACAGU-AAGUA
S. araneus (shrew)               CAA--------------------AAAUUUGAACCAAAACAUUCCGUUUACAUUUUAGACAGU-AAGUA
E. europaeus (hedgehog)          CAA--------------------AAAUUUGAACCAAAACAUUCCGUUUACAUUUUAGACAGU-AAGUA
C. l. familiaris (dog)           CAA--------------------AAAUUUGAACCAAAACAUUCCGUUUACAUUUUAGACAGU-AAGUA
F. catus (cat)                   CAA--------------------AAAUUUGAACCAAAACAUUCCGUUUACAUUUUAGACAGG-AAGUA
E. f. caballus (horse)           CAA--------------------AAAUUUGAACCAAAACAUUCCGUUUACAUUUUAGACAGU-AAGUA
B. taurus (cow)                  CAA--------------------AAAUUUGAACCAAAACAUUCCGUUUACAUUUUAGACAGU-AAGUA
D. rerio (zebrafish)             --------------------------------------------------------------------
L. africana (elephant)           CAA--------------------AAAUUUGAACCAAAACAUCCCGUUUACAUUUUAGACAGU-AAGUA
E. telfairi (tenrec)             CAA--------------------AAAUUUGAACCAAAACAUUCCGUUUACAUUUUAGACAGU-AAGUA
M. domestica (opossum)           AAC--------------------AAAUUUGAACCCAAACAUUCCGUUUCCAUUUUAGACAGUAAAGUA
O. anatinus (platypus)           -----------------------AAAUUUGAACC-AAACAUUCCGUUUACAUUUUAGACAGU-AAGUA
A. carolinensis (lizard)         ------------------------AAUUUGAACCAAAACAUUCCGUUUACAUUUUAGACAGU-AAGUA
X. tropicalis (frog)             -----------------------CAAUUUGAACCAAAACAUUCCGUUUACAUUUCACACAGU-A----
```

miR-30abcde/384-5p (*Mus musculus* BDNF 3’UTR nt pos 414-420)

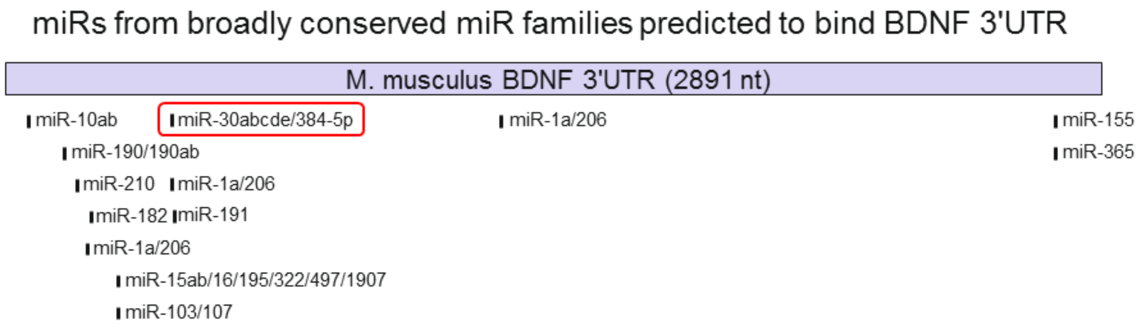

M. musculus (mouse)	AAUUGGAACCAAAACAUUCCGUUUACAUUUUAGACACU-AAGUAUCUU
H. sapiens (human)	AAUUUGAACCAAAACAUUCCGUUUACAUUUUAGACAGU-AAGUAUCUU
P. troglodytes (chimpanzee)	AAUUUGAACCAAAACAUUCCGUUUACAUUUUAGACAGU-AAGUAUCUU
M. mulatta (rhesus macaque)	AAUUUGAACCAAAACAUUCCGUUUACAUUUUAGACAGU-AAGUAUCUU
O. garnettii (bushbaby)	AAUUUGAACCCAAACAUUCCGUUUACAUUUAAGACAGU-AAGAUCCUC
T. belangeri (treeshrew)	AAUUUGAACCAAAACAUUCCGUUUCCAUUUUAGACAGU-ACGUAUCUU
R. norvegicus (rat)	AAUUUGAACCAAAACAUUCCGUUUACAUUUUAGACACU-AAGUAUCUU
C. porcellus (guinea pig)	AAUUUGAACCAAAACAUUCCGUUUACAUUUUAGACAGU-AAGUAUCUU
O. cuniculus (rabbit)	AAUUUGAACCAAAACAUUCCGUUUACAUUUUAGACAGU-AAGUAUCUC
S. araneus (shrew)	AAUUUGAACCAAAACAUUCCGUUUACAUUUUAGACAGU-AAGUAUCUU
E. europaeus (hedgehog)	AAUUUGAACCAAAACAUUCCGUUUACAUUUUAGACAGU-AAGUAUCUU
C. l. familiaris (dog)	AAUUUGAACCAAAACAUUCCGUUUACAUUUUAGACAGU-AAGUAUCUU
F. catus (cat)	AAUUUGAACCAAAACAUUCCGUUUACAUUUUAGACAGG-AAGUAUCUU
E. f. caballus (horse)	AAUUUGAACCAAAACAUUCCGUUUACAUUUUAGACAGU-AAGUAUCUU
B. taurus (cow)	AAUUUGAACCAAAACAUUCCGUUUACAUUUUAGACAGU-AAGUAUCUU
D. rerio (zebrafish)	--
L. africana (elephant)	AAUUUGAACCAAAACAUCCCGUUUACAUUUUAGACAGU-AAGUAUCUU
E. telfairi (tenrec)	AAUUUGAACCAAAACAUUCCGUUUACAUUUUAGACAGU-AAGUACCUG
M. domestica (opossum)	AAUUUGAACCCAAACAUUCCGUUUCCAUUUUAGACAGUAAAGUAUCUU
O. anatinus (platypus)	AAUUUGAACC-AAACAUUCCGUUUACAUUUUAGACAGU-AAGUAUCUU
A. carolinensis (lizard)	AAAUUGAACCAAAACAUUCCGUUUACAUUUUAGACAGU-AAGUAUCUU
X. tropicalis (frog)	AAUUUGAACCAAAACAUUCCGUUUACAUUUCACACAGU-A------UC

miR-1a/206, site 3 (*Mus musculus* BDNF 3'UTR nt pos 1306-1312)

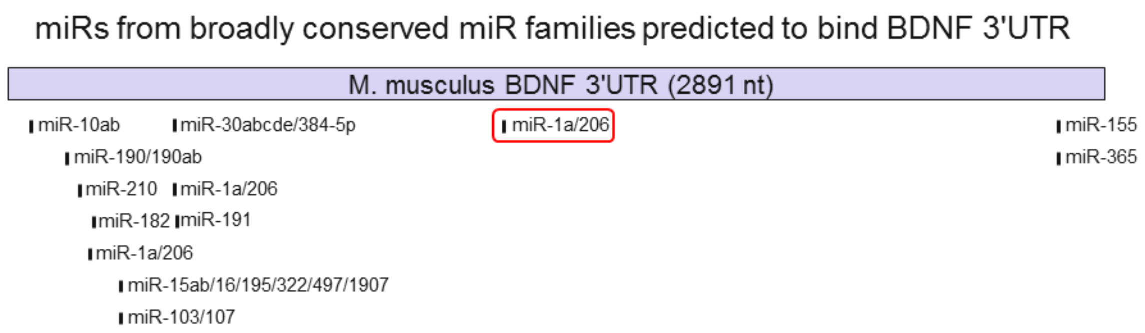

M. musculus (mouse)	GGUGGGGA-AUGGUA-CUUGAGACAUUCCU-AAAGGAA-GGC--------UCG--GAAGCA
H. sapiens (human)	GGUGGGGC-AUGGUA-UUUGAGACAUUCCA-AAACGAA-GGCC-------UCU--GAAGGA
P. troglodytes (chimpanzee)	GGUGGGGC-AUGGUA-UUUGAGACAUUCCA-AAACGAA-AGCC-------UCU--GAAGGA
M. mulatta (rhesus macaque)	GGUGGGGC-AUGGUA-UUUGAGACAUUCCA-AAAGGAA-GGCC-------UCU--GAAGGA
O. garnettii (bushbaby)	GGUGGGGA--UGGUA-CUUG-GACAUUCCA-AAAAAG-----C-------UCU--GAAGGA
T. belangeri (treeshrew)	GGUGGGGA-AUAGUA-CUUGAGACAUUCCA-CAAGGAA-AGCC-------UCU--GAAGGA
R. norvegicus (rat)	GGUGGGGA-AUGGUA-CUUGAGACAUUCCU-AAAGGAA-GGC--------UCG--GAAGCA
C. porcellus (guinea pig)	AGGGGGCG-AGGGUA-CUCGAGAUGCUCCU-ACAGGA---------------------GCA
O. cuniculus (rabbit)	GGUGGGGA-AUGGUA-UUUGAGACAUUCCA-AAAGGAA-GGUC-------UCU--UCAGGG
S. araneus (shrew)	GGUGGGGA-AUGAUA-CUUGAGACAUUCCA-AAAGGGA-GGCC-------UCU--GAAGGG
E. europaeus (hedgehog)	GGUUUGAA-CUCAUAGCUUGAGACAUUCCA-AGAGGAC-GGCC-------UCU--GGAGUG
C. l. familiaris (dog)	GGUGGGCA-ACGGUA-C-------AUUCCG-GAAGGGA-GGCC-------UCU--GAGGGA
F. catus (cat)	GGUGGGGA-AUGGUA-CUUGAGACAUUCCAAGUAGGA-GACC-------UUUUGAAGGAC
E. f. caballus (horse)	GCUGGCGA-GUGGUA-CUUGAGACAUUCCA-AAAGGGA-GGCC-------UCU--GAAGGA
B. taurus (cow)	GGUGGGGA-AGGGUA-CUUGAGACAUUCCA-GAAGGGA-GACC-------UCU--AAAGGA
D. rerio (zebrafish)	---
L. africana (elephant)	GGUAGGGA-AUGGUA-CUUGAGACAUUCCA-AAGGGGA-GGCC-------UCU--GAAGGA
E. telfairi (tenrec)	GGUAGGGA-AGGGGA-CGUGAGACAUUCCA-AAGGGGA-GGCC-------UCU--AAAGGA
M. domestica (opossum)	GGUGGAGAUGUGGCA-UUUGAGACAUUCAG-AAAGGAGCAGGA-------CCU--GAAGAA
O. anatinus (platypus)	--GGGCAU-GUGGUA-UUUGAGAAAUUCAG-AAAGAAA-UUCUGGGUAGAUGG--GAAGGG
A. carolinensis (lizard)	---
X. tropicalis (frog)	---

miR-365 (*Mus musculus* BDNF 3'UTR nt pos 2755-2761)

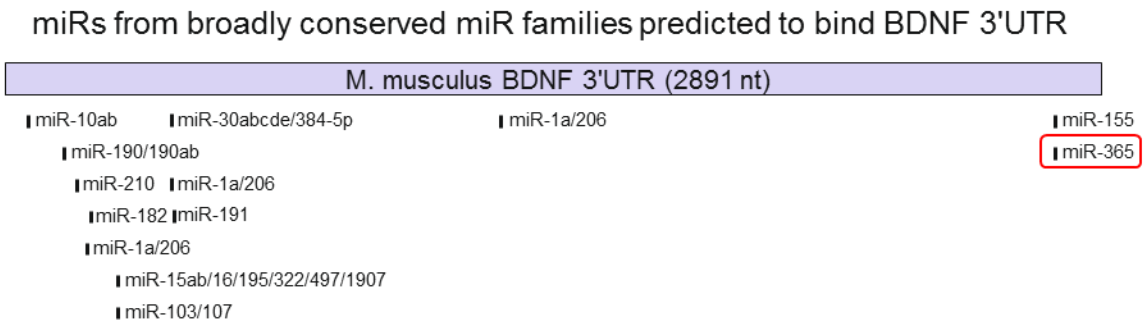

M. musculus (mouse)	UAUGUUAUUGAA-AAAA---UUGGCAUUAAAACUUAACCACAUCAGAAG
H. sapiens (human)	UAUGUUAUUGAA-AAAAA--UUGGCAUUAAAACUUAACCGCAUCAGAAG
P. troglodytes (chimpanzee)	UAUGUUAUUGAA-AAAAA--UUGGCAUUAAAACUUAACCGCAUCAGAAG
M. mulatta (rhesus macaque)	UAUGUUAUUGAA-AAAAA--UUGGCAUUAAAACUUAACCGCAUCAGAAG
O. garnettii (bushbaby)	UAUGUUAUUGAA-AAAAA--UUGGCAUUAAAACUUAACCGCAUCAGAAG
T. belangeri (treeshrew)	UAUGUA--UGAA-AAAA---UUGGCAUUAAA-CUUAACCGCAUCAGAAG
R. norvegicus (rat)	UAUGUUAUUGAA-AAAAA--UUGGCAUUAAAACUUAACCACAUCAGAAG
C. porcellus (guinea pig)	UAUGUUAUUGAA-AAAA---UUGGCAUUAAAAAUUAACCGCAUCAGAAG
O. cuniculus (rabbit)	UAUGUUAUUGAA-AAAAA--UUGGCAUUAAAACUUAACCGCAUCAGAAG
S. araneus (shrew)	UAUGUUAUUGAA-AAAAAA-UUGGCAUUAAAACUUAACCGCAUCAGAAG
E. europaeus (hedgehog)	UAUGUUAUUGAA-AAAAA--UUGGCAUUAAAACUUAACCUCAUCAGAAG
C. l. familiaris (dog)	UAUGUUAUUGAA-AAAAAAAUUGGCAUUAAAACUUAACCGCAUCAGAAG
F. catus (cat)	UAUGUUAUUGAA-AAAAA--UUGGCAUUAAAACUUAACCGCAUCAGAAG
E. f. caballus (horse)	UAUGUUAUUGAA-AAAA---UUGGCAUUAAAACUUAACCGCAUCAGAAG
B. taurus (cow)	UAUGUUCUUGAA-AAAAA--UUGGCAUUAAAACUUAAUCGCAUCAGAAG
D. rerio (zebrafish)	UAUGUUAUUGAA-AAAAA--UUGGCAUUAAAACUUAAUCGCAUCAGAAA
L. africana (elephant)	UAUGUUAUUGAAGAAAAA--UUGGCAUUAAAACUUAACCGCAUCAGAAG
E. telfairi (tenrec)	UAUGUUCUUGAAAAAAAA--UUGGCAUUAAAACUUAACCGCAUCAGAAG
M. domestica (opossum)	UCUGUUAUUGAG-AAAA---UUGGCAUUAAAACUUAAAAGCAUCAGAAU
O. anatinus (platypus)	UAUGUUAUUGAA-AAA----UUGGCAUUAAAACUUCAAAGCAUUAGAAU
A. carolinensis (lizard)	A---UUUUUGGA-AGAAU--CUGGCAUUAAAUCUUACAGGCAUCUGGUU
X. tropicalis (frog)	UAUGUUAUUGAA-AGAGA--UUGGCAUUUCAAAAUGGAAGCAUUUGAAA

miR-155 (*Mus musculus* BDNF 3'UTR nt pos 2756-2762)

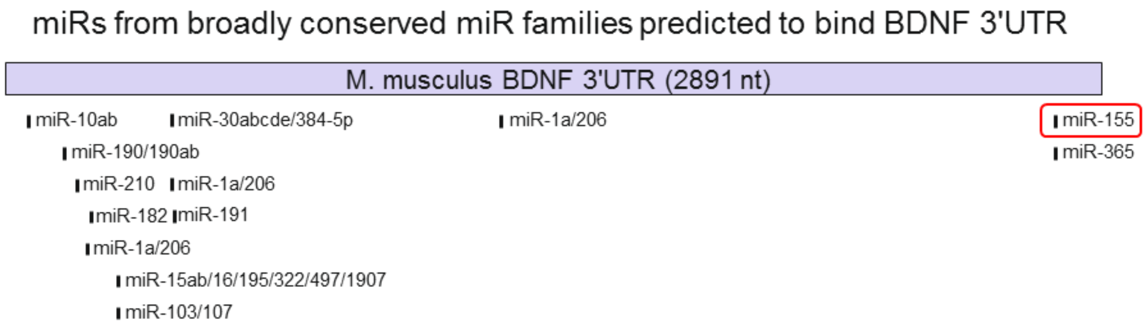

M. musculus (mouse)	AUGUUAUUGAA-AAAA---UUGGCAUUAAAACUUAACCACAUCAGAAGC
H. sapiens (human)	AUGUUAUUGAA-AAAAA--UUGGCAUUAAAACUUAACCGCAUCAGAAGC
P. troglodytes (chimpanzee)	AUGUUAUUGAA-AAAAA--UUGGCAUUAAAACUUAACCGCAUCAGAAGC
M. mulatta (rhesus macaque)	AUGUUAUUGAA-AAAAA--UUGGCAUUAAAACUUAACCGCAUCAGAAGC
O. garnettii (bushbaby)	AUGUUAUUGAA-AAAAA--UUGGCAUUAAAACUUAACCGCAUCAGAAGC
T. belangeri (treeshrew)	AUGUA--UGAA-AAAA---UUGGCAUUAAA-CUUAACCGCAUCAGAAGC
R. norvegicus (rat)	AUGUUAUUGAA-AAAAA--UUGGCAUUAAAACUUAACCACAUCAGAAGC
C. porcellus (guinea pig)	AUGUUAUUGAA-AAAA---UUGGCAUUAAAAAUUAACCGCAUCAGAAGC
O. cuniculus (rabbit)	AUGUUAUUGAA-AAAAA--UUGGCAUUAAAACUUAACCGCAUCAGAAGC
S. araneus (shrew)	AUGUUAUUGAA-AAAAAA-UUGGCAUUAAAACUUAACCGCAUCAGAAGC
E. europaeus (hedgehog)	AUGUUAUUGAA-AAAAA--UUGGCAUUAAAACUUAACCUCAUCAGAAGC
C. l. familiaris (dog)	AUGUUAUUGAA-AAAAAAAUUGGCAUUAAAACUUAACCGCAUCAGAAGC
F. catus (cat)	AUGUUAUUGAA-AAAAA--UUGGCAUUAAAACUUAACCGCAUCAGAAGC
E. f. caballus (horse)	AUGUUAUUGAA-AAAA---UUGGCAUUAAAACUUAACCGCAUCAGAAGC
B. taurus (cow)	AUGUUCUUGAA-AAAAA--UUGGCAUUAAAACUUAAUCGCAUCAGAAGC
D. rerio (zebrafish)	AUGUUAUUGAA-AAAAA--UUGGCAUUAAAACUUAAUCGCAUCAGAAAC
L. africana (elephant)	AUGUUAUUGAAGAAAAA--UUGGCAUUAAAACUUAACCGCAUCAGAAGC
E. telfairi (tenrec)	AUGUUCUUGAAAAAAAA--UUGGCAUUAAAACUUAACCGCAUCAGAAGC
M. domestica (opossum)	CUGUUAUUGAG-AAAA---UUGGCAUUAAAACUUAAAAGCAUCAGAAUC
O. anatinus (platypus)	AUGUUAUUGAA-AAA----UUGGCAUUAAAACUUCAAAGCAUUAGAAUC
A. carolinensis (lizard)	---UUUUUGGA-AGAAU--CUGGCAUUAAAUCUUACAGGCAUCUGGUUC
X. tropicalis (frog)	AUGUUAUUGAA-AGAGA--UUGGCAUUUCAAAAUGGAAGCAUUUGAAAC
