# Supplementary material for: miR-1, miR-10b, miR-155, and miR-191 are novel regulators of BDNF
Source: Cell Mol Life Sci. 2014 May 8;71(22):4443–56. doi: 10.1007/s00018-014-1628-x (PMC4207943; doi:10.1007/s00018-014-1628-x)
Supplement: Supplementary file 4 — Supplementary material 4 (PDF 399 kb) [file 18_2014_1628_MOESM4_ESM.pdf]

miR-1, miR-10b, miR-155 and miR-191 are novel regulators of BDNF

Cellular and Molecular Life Sciences

Kärt Varendi, Anmol Kumar, Mari-Anne Härma and Jaan-Olle Andressoo*
Institute of Biotechnology, University of Helsinki, 00014, Finland
*To whom correspondence should be addressed. Tel. +358 50 358 1213; E-mail: jaan-olle.andressoo@helsinki.fi

Online resource 3

A. Broadly conserved microRNA families	Seed + m8
let-7/98/4458/4500	GAGGUAG
miR-1ab/206/613	GGAAUGU
miR-7/7ab	GGAAGAC
miR-9/9ab	CUUUGGU
miR-10abc/10a-5p	ACCCUGU
miR-15abc/16/16abc/195/322/424/497/1907	AGCAGCA
miR-17/17-5p/20ab/20b-5p/93/106ab/427/518a-3p/519d	AAAGUGC
miR-18ab/4735-3p	AAGGUGC
miR-19ab	GUGCAAA
miR-21/590-5p	AGCUUAU
miR-22/22-3p	AGCUGCC
miR-23abc/23b-3p	UCACAUU
miR-24/24ab/24-3p	GGCUCAG
miR-25/32/92abc/363/363-3p/367	AUUGCAC
miR-26ab/1297/4465	UCAAGUA
miR-27abc/27a-3p	UCACAGU
miR-29abcd	AGCACCA
miR-30abcdef/30abe-5p/384-5p	GUAAACA
miR-31	GGCAAGA
miR-33a-3p/365/365-3p	AAUGCCC
miR-33ab/33-5p	UGCAUUG
miR-34ac/34bc-5p/449abc/449c-5p	GGCAGUG
miR-93/93a/105/106a/291a-3p/294/295/302abcde/372/373/428/519a/520be/520acd-3p/1378/1420ac	AAGUGCU
miR-96/507/1271	UUGGCAC
miR-99ab/100	ACCCGUA
miR-101/101ab	ACAGUAC
miR-103a/107/107ab	GCAGCAU
miR-122/122a/1352	GGAGUGU
miR-124/124ab/506	AAGGCAC
miR-125a-5p/125b-5p/351/670/4319	CCCUGAG
miR-126-3p	CGUACCG
miR-128/128ab	CACAGUG
miR-129-5p/129ab-5p	UUUUUGC
miR-130ac/301ab/301b/301b-3p/454/721/4295/3666	AGUGCAA
miR-132/212/212-3p	AACAGUC
miR-133abc	UUGGUCC
miR-135ab/135a-5p	AUGGCUU
miR-137/137ab	UAUUGCU
miR-138/138ab	GCUGGUG
miR-139-5p	CUACAGU

miR-140/140-5p/876-3p/1244	AGUGGUU
miR-141/200a	AACACUG
miR-142-3p	GUAGUGU
miR-143/1721/4770	GAGAUGA
miR-144	ACAGUAU
miR-145	UCCAGUU
miR-146ac/146b-5p	GAGAACU
miR-148ab-3p/152	CAGUGCA
miR-150/5127	CUCCCAA
miR-153	UGCAUAG
miR-155	UAAUGCU
miR-181abcd/4262	ACAUUCA
miR-182	UUGGCAA
miR-183	AUGGCAC
miR-184	GGACGGA
miR-187	CGUGUCU
miR-190/190ab	GAUAUGU
miR-191	AACGGAA
miR-192/215	UGACCUA
miR-193/193b/193a-3p	ACUGGCC
miR-194	GUAACAG
miR-196abc	AGGUAGU
miR-199ab-5p	CCAGUGU
miR-200bc/429/548a	AAUACUG
miR-203	UGAAAUG
miR-204/204b/211	UCCCUUU
miR-205/205ab	CCUUCAU
miR-208ab/208ab-3p	UAAGACG
miR-210	UGUGCGU
miR-214/761/3619-5p	CAGCAGG
miR-216a	AAUCUCA
miR-216b/216b-5p	AAUCUCU
miR-217	ACUGCAU
miR-218/218a	UGUGCUU
miR-219-5p/508/508-3p/4782-3p	GAUUGUC
miR-221/222/222ab/1928	GCUACAU
miR-223	GUCAGUU
miR-338/338-3p	CCAGCAU
miR-375	UUGUUCG
miR-383	GAUCAGA
miR-425/425-5p/489	AUGACAC
miR-451	AACCGUU
miR-455-5p	AUGUGCC
miR-490-3p	AACCUGG
miR-499-5p	UAAGACU
miR-503	AGCAGCG
miR-551a	CGACCCA

B. List of miRs from broadly conserved families

let-7		miR-138a		miR-193		miR-222a		miR-338		miR-503
miR-100		miR-138b		miR-193a-3p		miR-222b		miR-338-3p		miR-506
miR-101		miR-139-5p		miR-193b		miR-223		miR-33a		miR-507
miR-101a		miR-140		miR-194		miR-22-3p		miR-33a-3p		miR-508
miR-101b		miR-140-5p		miR-195		miR-23a		miR-33b		miR-508-3p
miR-103		miR-141		miR-196a		miR-23b		miR-34a		miR-5127
miR-103a		miR-1420a		miR-196b		miR-23b-3p		miR-34b-5p		miR-518-3p
miR-105		miR-1420c		miR-196c		miR-23c		miR-34c		miR-519a
miR-106a		miR-142-3p		miR-199a-5p		miR-24		miR-34c-5p		miR-519d
miR-106b		miR-143		miR-199b-5p		miR-24-3p		miR-351		miR-520a-3p
miR-107		miR-144		miR-19a		miR-24a		miR-3619-5p		miR-520b
miR-107a		miR-145		miR-19b		miR-24b		miR-363		miR-520c-3p
miR-107b		miR-146a		miR-1a		miR-25		miR-363-3p		miR-520d-3p
miR-10a		miR-146b-5p		miR-1b		miR-26a		miR-365		miR-520e
miR-10b		miR-146c		miR-200a		miR-26b		miR-365-3p		miR-548a
miR-10c		miR-148a-3p		miR-200b		miR-27a		miR-3666		miR-551a
miR-122		miR-148b-3p		miR-200c		miR-27a-3p		miR-367		miR-590-5p
miR-122a		miR-150		miR-203		miR-27b		miR-372		miR-613
miR-124		miR-152		miR-204		miR-27c		miR-373		miR-670
miR-1244		miR-153		miR-204b		miR-291a-3p		miR-375		miR-7
miR-124a		miR-155		miR-205		miR-294		miR-383		miR-721
miR-124b		miR-15a		miR-205a		miR-295		miR-384-5p		miR-761
miR-125a-5p		miR-15b		miR-205b		miR-29a		miR-424		miR-7a
miR-125b-5p		miR-15c		miR-206		miR-29b		miR-425		miR-7b
miR-126-3p		miR-16		miR-208a		miR-29c		miR-425-5p		miR-876-3p
miR-1271		miR-16a		miR-208a-3p		miR-29d		miR-4262		miR-9
miR-128		miR-16b		miR-208b		miR-301a		miR-427		miR-92a
miR-128a		miR-16c		miR-208b-3p		miR-301b		miR-428		miR-92b
miR-128b		miR-17		miR-20a		miR-301b		miR-429		miR-92c
miR-129-5p		miR-1721		miR-20b		miR-301b-3p		miR-4295		miR-93
miR-1297		miR-17-5p		miR-20b-5p		miR-302a		miR-4319		miR-93
miR-129a-5p		miR-181a		miR-21		miR-302b		miR-4458		miR-93a
miR-129b-5p		miR-181b		miR-210		miR-302c		miR-4465		miR-96
miR-130a		miR-181c		miR-211		miR-302d		miR-449a		miR-98
miR-130c		miR-181d		miR-212		miR-302e		miR-449b		miR-99a
miR-132		miR-182		miR-212-3p		miR-30a		miR-449c		miR-99b
miR-133a		miR-183		miR-214		miR-30a-5p		miR-449c-5p		miR-9a
miR-133b		miR-184		miR-215		miR-30b		miR-4500		miR-9b
miR-133c		miR-187		miR-216a		miR-30b-5p		miR-451		
miR-1352		miR-18a		miR-216b		miR-30c		miR-454		
miR-135a		miR-18b		miR-216b-5p		miR-30d		miR-455-5p		
miR-135a-5p		miR-190		miR-217		miR-30e		miR-4735-3p		
miR-135b		miR-1907		miR-218		miR-30e-5p		miR-4770		
miR-137		miR-190a		miR-218a		miR-30f		miR-4782-3p		
miR-1378		miR-190b		miR-219-5p		miR-31		miR-489		
miR-137a		miR-191		miR-22		miR-32		miR-490-3p		
miR-137b		miR-192		miR-221		miR-322		miR-497		
miR-138		miR-1928		miR-222		miR-33-5p		miR-499-5p		

C. miR binding sites within Mus musculus BDNF 3'UTR predicted with different bioinformatics tools

microRNA	Position	TargetScan	PITA ddG<-10	PITA ddG<0	miRanda	PicTar
mmu-miR-144	22			+		
mmu-miR-24	25			+		
mmu-miR-10a	59	+		+	+	+
mmu-miR-10b	59	+		+	+	+
mmu-miR-191	72			+		
mmu-miR-196a	99			+		
mmu-miR-144	121			+		
mmu-miR-101a	126			+		
mmu-miR-101b	126			+		
mmu-miR-141	129			+		
mmu-miR-200a	129			+		
mmu-miR-429	129			+		
mmu-miR-1	139			+		
mmu-miR-206	139			+		
mmu-miR-33	140			+		
mmu-miR-190	155	+	+	+	+	
mmu-miR-190b	155	+	+	+	+	
mmu-miR-135a	179			+		
mmu-miR-135b	179			+		
mmu-miR-19a	185			+		
mmu-miR-19b	185			+		
mmu-miR-210	189	+		+	+	
mmu-miR-375	189			+		
mmu-miR-1	208			+		
mmu-miR-206	208			+		
mmu-miR-203	209			+		
mmu-miR-130a	211			+		
mmu-miR-130b	211			+		
mmu-miR-1	213	+		+	+	+
mmu-miR-206	213	+	+	+	+	+
mmu-miR-141	224			+		
mmu-miR-200a	224			+		
mmu-miR-221	226			+		
mmu-miR-455	232			+		
mmu-miR-322	237			+		
mmu-miR-322	240			+		
mmu-miR-194	242			+		
mmu-miR-503	243		+	+		
mmu-miR-182	245	+	+	+	+	+
mmu-miR-96	245			+		
mmu-miR-129-5p	262				+	
mmu-miR-499	267			+		
mmu-miR-19a	289			+		
mmu-miR-19b	289			+		
mmu-miR-130a	290			+		
mmu-miR-130b	290			+		
mmu-miR-301a	290			+		

microRNA	Position	TargetScan	PITA ddG<-10	PITA ddG<0	miRanda	PicTar
mmu-miR-301b	290			+		
mmu-miR-721	290			+		
mmu-miR-214	293			+		
mmu-miR-103	294	+		+	+	+
mmu-miR-107	294	+		+	+	+
mmu-miR-15a	295	+		+	+	+
mmu-miR-15b	295	+		+	+	+
mmu-miR-16	295	+	+	+	+	+
mmu-miR-195	295	+		+	+	+
mmu-miR-29a	295			+		
mmu-miR-29b	295			+		
mmu-miR-29c	295			+		
mmu-miR-322	295	+		+	+	
mmu-miR-497	295	+	+	+	+	
mmu-miR-503	295			+	+	
mmu-miR-128	304			+		
mmu-miR-27a	305			+		
mmu-miR-27b	305			+		
mmu-miR-182	306			+		
mmu-miR-455	309			+		
mmu-miR-590-5p	319			+		
mmu-miR-223	322			+		
mmu-miR-34a	322			+		
mmu-miR-34b-5p	322			+		
mmu-miR-34c	322			+		
mmu-miR-449a	322			+		
mmu-miR-449b	322			+		
mmu-miR-190	342			+		
mmu-miR-190b	342			+		
mmu-miR-129-5p	356				+	
mmu-miR-129-5p	363				+	
mmu-miR-129-5p	377				+	
mmu-miR-129-5p	384				+	
mmu-miR-199a-5p	393			+		
mmu-miR-145	394			+		
mmu-miR-133a	399			+		
mmu-miR-133b	399			+		
mmu-miR-9	401			+		
mmu-miR-1	407	+		+	+	+
mmu-miR-122	407			+		
mmu-miR-206	407	+		+	+	+
mmu-miR-203	408			+		
mmu-miR-191	410	+		+	+	
mmu-miR-30a	414	+				
mmu-miR-30b	414	+				
mmu-miR-30c	414	+				
mmu-miR-30d	414	+				

microRNA	Position	TargetScan	PITA ddG<-10	PITA ddG<0	miRanda	PicTar
mmu-miR-30e	414	+				+
mmu-miR-384-5p	414	+				
mmu-miR-203	419			+		
mmu-miR-139-5p	420			+		
mmu-miR-142-3p	427			+		
mmu-miR-153	430			+		
mmu-miR-196a	433			+		
mmu-miR-196b	433			+		
mmu-miR-146a	451			+		
mmu-miR-146b	451			+		
mmu-miR-295	451			+		
mmu-miR-101a	452			+		
mmu-miR-101b	452			+		
mmu-miR-106a	452			+		
mmu-miR-106b	452			+		
mmu-miR-17	452			+		
mmu-miR-20a	452			+		
mmu-miR-20b	452			+		
mmu-miR-93	452			+		
mmu-miR-322	463			+		
mmu-miR-383	477			+		
mmu-miR-141	482			+		
mmu-miR-200a	482			+		
mmu-miR-155	489			+		
mmu-miR-33	499			+		
mmu-miR-449b	499			+		
mmu-miR-29a	507			+		
mmu-miR-29b	507			+		
mmu-miR-29c	507			+		
mmu-miR-217	509			+		
mmu-miR-15a	510			+		
mmu-miR-15b	510			+		
mmu-miR-16	510			+		
mmu-miR-195	510			+		
mmu-miR-322	510			+		
mmu-miR-497	510			+		
mmu-miR-489	515			+		
mmu-miR-130a	521			+		
mmu-miR-130b	521			+		
mmu-miR-187	537			+		
mmu-miR-191	563			+		
mmu-miR-144	583			+		
mmu-miR-384-5p	604			+		
mmu-miR-206	653			+		
mmu-miR-490	657			+		
mmu-miR-181a	680			+		
mmu-miR-30a	695		+	+		

microRNA	Position	TargetScan	PITA ddG<-10	PITA ddG<0	miRanda	PicTar
mmu-miR-30b	695			+		
mmu-miR-30c	695			+		
mmu-miR-30d	695			+		
mmu-miR-30e	695			+		
mmu-miR-384-5p	695			+		
mmu-miR-19a	697			+		
mmu-miR-19b	697			+		
mmu-miR-129-5p	700			+		
mmu-miR-145	717			+		
mmu-miR-205	736			+		
mmu-miR-122	761		+	+	+	
mmu-miR-34a	767		+	+	+	
mmu-miR-34b-5p	767		+	+	+	
mmu-miR-34c	767		+	+	+	
mmu-miR-449a	767		+	+	+	
mmu-miR-449b	767		+	+	+	
mmu-miR-449c	767		+	+	+	
mmu-miR-221	778			+		
mmu-miR-222	778			+		
mmu-miR-143	780			+		
mmu-miR-122	803			+		
mmu-miR-122	805			+		
mmu-miR-7a	806			+		
mmu-miR-7b	806			+		
mmu-miR-761	807		+	+		
mmu-miR-215	817			+		
mmu-miR-551b	817			+		
mmu-miR-670	821			+		
mmu-miR-138	847		+	+		
mmu-miR-291a-3p	851			+		
mmu-miR-294	851			+		
mmu-miR-295	851			+		
mmu-miR-302a	851			+		
mmu-miR-302b	851			+		
mmu-miR-302d	851			+		
mmu-miR-105	852			+		
mmu-miR-106a	852			+		
mmu-miR-106b	852			+		
mmu-miR-17	852			+		
mmu-miR-20a	852			+		
mmu-miR-20b	852			+		
mmu-miR-93	852			+		
mmu-miR-19a	855			+		
mmu-miR-19b	855			+		
mmu-miR-218	863			+		
mmu-miR-7a	870			+		
mmu-miR-7b	870			+		

microRNA	Position	TargetScan	PITA ddG<-10	PITA ddG<0	miRanda	PicTar
mmu-miR-184	871			+		
mmu-miR-184	875			+		
mmu-let-7e	878			+		
mmu-miR-184	879			+		
mmu-miR-19a	934			+		
mmu-miR-19b	934			+		
mmu-miR-106a	939			+		
mmu-miR-106b	939			+		
mmu-miR-124	939			+		
mmu-miR-17	939			+		
mmu-miR-20a	939			+		
mmu-miR-20b	939			+		
mmu-miR-93	939			+		
mmu-miR-130a	952			+		
mmu-miR-130b	952			+		
mmu-miR-301a	952			+		
mmu-miR-301b	952			+		
mmu-miR-721	952			+		
mmu-let-7a	957			+		
mmu-let-7b	957			+		
mmu-let-7c	957			+		
mmu-let-7d	957			+		
mmu-let-7f	957			+		
mmu-let-7g	957			+		
mmu-let-7i	957			+		
mmu-miR-98	957			+		
mmu-miR-503	958			+		
mmu-miR-375	966			+		
mmu-miR-223	968			+		
mmu-miR-375	974			+		
mmu-miR-181b	981			+		
mmu-miR-181d	981			+		
mmu-miR-183	984			+		
mmu-miR-425	984			+	+	
mmu-miR-489	984			+	+	
mmu-miR-670	1001			+		
mmu-miR-190b	1057			+		
mmu-miR-135a	1074			+		
mmu-miR-135b	1074			+		
mmu-miR-145	1093			+		
mmu-miR-25	1095			+		
mmu-miR-367	1095			+		
mmu-miR-721	1095			+		
mmu-miR-92a	1095			+		
mmu-miR-92b	1095			+		
mmu-miR-141	1111			+	+	
mmu-miR-200a	1111			+	+	

microRNA	Position	TargetScan	PITA ddG<-10	PITA ddG<0	miRanda	PicTar
mmu-miR-200b	1111			+		
mmu-miR-200c	1111			+		
mmu-miR-429	1111		+	+		
mmu-miR-384-5p	1114			+		
mmu-miR-19a	1116			+		
mmu-miR-19b	1116			+		
mmu-miR-384-5p	1134			+		
mmu-miR-216b	1163			+		
mmu-miR-133a	1165			+		
mmu-miR-133b	1165			+		
mmu-miR-142-3p	1171			+		
mmu-miR-34a	1172			+	+	
mmu-miR-34b-5p	1172			+	+	
mmu-miR-34c	1172			+	+	
mmu-miR-449a	1172			+	+	
mmu-miR-449b	1172			+	+	
mmu-miR-449c	1172			+	+	
mmu-miR-145	1184			+		
mmu-miR-761	1200			+		
mmu-miR-221	1214			+		
mmu-miR-29a	1215			+		
mmu-miR-29b	1215			+		
mmu-miR-29c	1215			+		
mmu-miR-22	1237			+		
mmu-miR-205	1241			+		
mmu-miR-25	1241			+		
mmu-miR-367	1241			+		
mmu-miR-92a	1241			+		
mmu-miR-92b	1241			+		
mmu-miR-383	1255			+		
mmu-miR-26a	1298			+		
mmu-miR-26b	1298			+		
mmu-miR-1	1306	+		+		+
mmu-miR-122	1306			+		
mmu-miR-206	1306	+		+		+
mmu-miR-182	1309			+		
mmu-miR-204	1314			+		
mmu-miR-211	1314			+		
mmu-miR-205	1316			+		
mmu-miR-302c	1328		+	+		
mmu-miR-351	1336			+		
mmu-miR-103	1354			+		
mmu-miR-107	1354			+		
mmu-miR-222	1357			+		
mmu-miR-425	1359			+		
mmu-miR-96	1359			+		
mmu-miR-214	1376			+		

microRNA	Position	TargetScan	PITA ddG<-10	PITA ddG<0	miRanda	PicTar
mmu-miR-503	1378			+		
mmu-miR-383	1379			+		
mmu-miR-196a	1388			+		
mmu-miR-196b	1388			+		
mmu-miR-96	1389			+		
mmu-let-7e	1393			+		
mmu-miR-142-3p	1395			+		
mmu-miR-206	1395			+		
mmu-miR-34a	1396			+		
mmu-miR-34b-5p	1396			+		
mmu-miR-34c	1396			+		
mmu-miR-449a	1396			+		
mmu-miR-449b	1396			+		
mmu-miR-196a	1397			+		
mmu-miR-196b	1397			+		
mmu-let-7a	1398			+		
mmu-let-7b	1398			+		
mmu-let-7c	1398			+		
mmu-let-7d	1398			+		
mmu-let-7f	1398			+		
mmu-let-7g	1398			+		
mmu-let-7i	1398			+		
mmu-miR-98	1398			+		
mmu-miR-142-3p	1405			+		
mmu-miR-141	1406			+		
mmu-miR-200a	1406			+		
mmu-miR-33	1406			+		
mmu-miR-34a	1406			+		
mmu-miR-34b-5p	1406			+		
mmu-miR-34c	1406			+		
mmu-miR-429	1406			+		
mmu-miR-449a	1406			+		
mmu-miR-449b	1406			+		
mmu-miR-146a	1407			+		
mmu-miR-146b	1407			+		
mmu-miR-96	1408			+		
mmu-miR-216b	1415			+		
mmu-miR-146a	1418			+		
mmu-miR-146b	1418			+		
mmu-miR-140	1433			+		
mmu-miR-122	1437			+		
mmu-miR-184	1459			+		
mmu-miR-182	1468			+		
mmu-miR-128	1477			+		
mmu-miR-384-5p	1480			+		
mmu-miR-137	1531				+	
mmu-miR-33	1545			+		

microRNA	Position	TargetScan	PITA ddG<-10	PITA ddG<0	miRanda	PicTar
mmu-miR-103	1547			+		
mmu-miR-107	1547			+		
mmu-miR-221	1547			+		
mmu-miR-30a	1565			+		
mmu-miR-30b	1565			+		
mmu-miR-30c	1565			+		
mmu-miR-30d	1565			+		
mmu-miR-30e	1565			+		
mmu-miR-384-5p	1565		+	+		
mmu-miR-322	1569			+		
mmu-miR-19a	1587			+		
mmu-miR-19b	1587			+		
mmu-miR-216a	1594			+		
mmu-miR-351	1605			+		
mmu-miR-670	1606			+		
mmu-miR-490	1615			+		
mmu-miR-384-5p	1621			+		
mmu-miR-384-5p	1629			+		
mmu-miR-204	1653			+		
mmu-miR-211	1653			+		
mmu-miR-375	1671			+		
mmu-miR-375	1675			+		
mmu-miR-375	1679			+		
mmu-miR-375	1683			+		
mmu-miR-9	1691			+		
mmu-miR-670	1711			+		
mmu-miR-218	1713			+		
mmu-miR-590-5p	1720			+		
mmu-miR-32	1722			+		
mmu-miR-449c	1725			+		
mmu-miR-193b	1738			+		
mmu-miR-187	1751		+	+		
mmu-miR-190	1753			+		
mmu-miR-190b	1753			+		
mmu-miR-29a	1761			+		
mmu-miR-29b	1761			+		
mmu-miR-29c	1761			+		
mmu-miR-124	1763			+		
mmu-miR-128	1770			+	+	
mmu-miR-139-5p	1771			+		
mmu-miR-27a	1771			+	+	
mmu-miR-27b	1771			+	+	
mmu-miR-187	1781			+		
mmu-miR-218	1785			+		
mmu-miR-199a-5p	1789			+		
mmu-miR-103	1801			+		
mmu-miR-107	1801			+		

microRNA	Position	TargetScan	PITA ddG<-10	PITA ddG<0	miRanda	PicTar
mmu-miR-31	1802			+		
mmu-miR-351	1808			+		
mmu-miR-670	1809			+		
mmu-miR-205	1812			+		
mmu-miR-183	1851			+		
mmu-miR-106a	1860			+		
mmu-miR-106b	1860			+		
mmu-miR-144	1860			+		
mmu-miR-17	1860			+		
mmu-miR-20a	1860			+		
mmu-miR-20b	1860			+		
mmu-miR-93	1860			+		
mmu-miR-205	1902			+		
mmu-miR-181b	1903			+		
mmu-miR-181d	1903			+		
mmu-miR-23a	1914			+		
mmu-miR-490	1946			+		
mmu-miR-181a	1964			+		
mmu-miR-181b	1964			+		
mmu-miR-181c	1964			+		
mmu-miR-181d	1964			+		
mmu-miR-194	1976			+		
mmu-miR-30a	1976			+		
mmu-miR-30b	1976			+		
mmu-miR-30c	1976			+		
mmu-miR-30d	1976			+		
mmu-miR-30e	1976			+		
mmu-miR-122	1981			+		
mmu-miR-761	1983		+	+		
mmu-miR-375	2025			+		
mmu-miR-302a	2031			+		
mmu-miR-143	2037			+		
mmu-miR-18b	2037			+		
mmu-miR-208b	2038			+		
mmu-miR-125a-5p	2041			+		
mmu-miR-125b-5p	2041			+		
mmu-miR-128	2074			+		
mmu-miR-503	2074			+		
mmu-miR-27a	2075			+		
mmu-miR-27b	2075			+		
mmu-miR-194	2076			+		
mmu-miR-125a-5p	2082			+	+	
mmu-miR-125b-5p	2082			+	+	
mmu-miR-351	2082			+	+	
mmu-miR-670	2083		+	+		
mmu-miR-216b	2085			+		
mmu-miR-205	2090		+	+		

microRNA	Position	TargetScan	PITA ddG<-10	PITA ddG<0	miRanda	PicTar
mmu-miR-187	2094		+	+		
mmu-miR-138	2097			+		
mmu-miR-138	2100			+		
mmu-miR-383	2120			+		
mmu-miR-124	2131			+		
mmu-miR-124	2148			+		
mmu-miR-140	2161			+		
mmu-miR-876-3p	2161			+	+	
mmu-miR-590-5p	2170			+		
mmu-miR-103	2172			+		
mmu-miR-107	2172			+		
mmu-miR-132	2173			+		
mmu-miR-15a	2173			+		
mmu-miR-15b	2173			+		
mmu-miR-16	2173			+		
mmu-miR-191	2173			+		
mmu-miR-195	2173			+		
mmu-miR-212	2173			+		
mmu-miR-29b	2173			+		
mmu-miR-322	2173			+		
mmu-miR-497	2173			+		
mmu-miR-194	2175			+		
mmu-miR-221	2193			+	+	
mmu-miR-222	2193			+	+	
mmu-miR-132	2203			+		
mmu-miR-212	2203			+		
mmu-miR-124	2212			+		
mmu-miR-183	2212			+		
mmu-miR-32	2212			+		
mmu-miR-15a	2213			+		
mmu-miR-15b	2213			+		
mmu-miR-16	2213			+		
mmu-miR-191	2213			+		
mmu-miR-195	2213			+		
mmu-miR-497	2213			+		
mmu-miR-223	2225			+		
mmu-miR-425	2234			+		
mmu-miR-208a	2241			+		
mmu-miR-208b	2241			+		
mmu-miR-190	2250			+		
mmu-miR-190b	2250			+		
mmu-miR-670	2256			+		
mmu-miR-490	2280			+		
mmu-miR-30e	2283			+		
mmu-miR-22	2294			+		+
mmu-miR-322	2294			+		
mmu-miR-18a	2295		+	+		

microRNA	Position	TargetScan	PITA ddG<-10	PITA ddG<0	miRanda	PicTar
mmu-miR-18b	2295			+		
mmu-miR-1	2301			+		
mmu-miR-206	2301			+		
mmu-miR-27a	2303			+		
mmu-miR-375	2325			+		
mmu-miR-9	2328			+		
mmu-miR-204	2330			+		
mmu-miR-211	2330			+		
mmu-miR-122	2345			+		
mmu-miR-429	2354			+		
mmu-miR-101a	2356		+	+		
mmu-miR-101b	2356		+	+		
mmu-miR-103	2356			+		
mmu-miR-107	2356			+		
mmu-miR-449c	2357			+		
mmu-miR-96	2365			+		
mmu-miR-193	2366			+		
mmu-miR-193b	2366			+		
mmu-miR-193	2371			+		
mmu-miR-132	2372			+		
mmu-miR-212	2372			+		
mmu-miR-138	2397		+	+		
mmu-miR-105	2402			+		
mmu-miR-193	2412			+		
mmu-miR-193b	2412		+	+		
mmu-miR-187	2440		+	+		
mmu-miR-196a	2452			+		
mmu-miR-196b	2452			+		
mmu-miR-150	2460			+		
mmu-miR-30a	2471			+		
mmu-miR-30b	2471			+		
mmu-miR-30c	2471			+		
mmu-miR-30d	2471			+		
mmu-miR-216a	2479			+	+	
mmu-miR-26a	2486			+		
mmu-miR-26b	2486			+		
mmu-miR-876-3p	2496			+		
mmu-miR-191	2510			+		
mmu-miR-383	2511			+		
mmu-miR-490	2520			+		
mmu-miR-101a	2524			+		
mmu-miR-101b	2524			+		
mmu-miR-15a	2525			+		
mmu-miR-15b	2525			+		
mmu-miR-16	2525			+		
mmu-miR-195	2525			+		
mmu-miR-497	2525			+		

microRNA	Position	TargetScan	PITA ddG<-10	PITA ddG<0	miRanda	PicTar
mmu-miR-383	2526			+		
mmu-miR-144	2529			+		
mmu-miR-15a	2530			+		
mmu-miR-15b	2530			+		
mmu-miR-16	2530			+		
mmu-miR-195	2530			+		
mmu-miR-497	2530			+		
mmu-miR-383	2531			+		
mmu-miR-144	2534			+		
mmu-miR-15a	2535			+		
mmu-miR-15b	2535			+		
mmu-miR-16	2535			+		
mmu-miR-195	2535		+	+		
mmu-miR-497	2535			+		
mmu-miR-383	2536			+		
mmu-miR-144	2539			+		
mmu-miR-15a	2540			+		
mmu-miR-15b	2540			+		
mmu-miR-16	2540			+		
mmu-miR-195	2540			+		
mmu-miR-497	2540			+		
mmu-miR-383	2541			+		
mmu-miR-144	2544			+		
mmu-miR-15a	2545			+		
mmu-miR-15b	2545			+		
mmu-miR-16	2545			+		
mmu-miR-195	2545			+		
mmu-miR-497	2545			+		
mmu-miR-383	2546			+		
mmu-miR-208a	2554			+		
mmu-miR-208b	2554			+		
mmu-miR-135a	2561			+		
mmu-miR-135b	2561			+		
mmu-miR-499	2562			+		
mmu-miR-7a	2563			+		
mmu-miR-7b	2563			+		
mmu-miR-15a	2564			+		
mmu-miR-15b	2564			+		
mmu-miR-16	2564			+		
mmu-miR-195	2564			+		
mmu-miR-497	2564			+		
mmu-miR-214	2565		+	+		
mmu-miR-322	2567			+		
mmu-miR-384-5p	2580			+		
mmu-miR-150	2582			+		
mmu-miR-129-5p	2585			+		
mmu-miR-153	2596			+		

microRNA	Position	TargetScan	PITA ddG<-10	PITA ddG<0	miRanda	PicTar
mmu-miR-22	2604			+		
mmu-miR-214	2613			+		
mmu-miR-194	2614			+		
mmu-miR-15a	2615			+		
mmu-miR-497	2615			+		
mmu-miR-145	2644			+		
mmu-miR-181b	2653			+		
mmu-miR-181d	2653			+		
mmu-miR-146a	2669			+		
mmu-miR-146b	2669			+		
mmu-miR-190	2686			+		
mmu-miR-190b	2686			+		
mmu-miR-429	2689			+		
mmu-miR-30a	2737			+		
mmu-miR-30d	2737			+		
mmu-miR-30e	2737			+		
mmu-miR-145	2763			+		
mmu-miR-19a	2765			+		
mmu-miR-19b	2765			+		
mmu-miR-365	2766	+			+	
mmu-miR-155	2767	+		+	+	
mmu-miR-218	2780			+		
mmu-miR-138	2785			+		
mmu-miR-214	2794			+		
mmu-miR-182	2798			+		
mmu-miR-10a	2806			+		
mmu-miR-10b	2806			+		
mmu-miR-490	2806			+	+	
mmu-miR-221	2821			+		
mmu-miR-190	2833			+		
mmu-miR-190b	2833			+		
mmu-miR-190b	2840			+		
mmu-miR-499	2845			+		
mmu-miR-93	2858			+		
mmu-miR-182	2882			+		
