# Supplementary material for: miR-1, miR-10b, miR-155, and miR-191 are novel regulators of BDNF
Source: Cell Mol Life Sci. 2014 May 8;71(22):4443–56. doi: 10.1007/s00018-014-1628-x (PMC4207943; doi:10.1007/s00018-014-1628-x)

miR-1, miR-10b, miR-155 and miR-191 are novel regulators of BDNF

Cellular and Molecular Life Sciences

Kärt Varendi, Anmol Kumar, Mari-Anne Härma and Jaan-Olle Andressoo*
Institute of Biotechnology, University of Helsinki, 00014, Finland
*To whom correspondence should be addressed. Tel: +358 50 358 1213; E-mail: jaan-olle.andressoo@helsinki.fi

Online resource 4

a BDNF mRNA levels

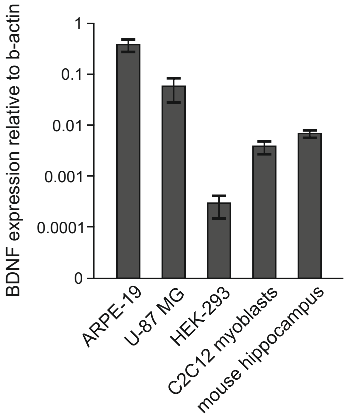

b Conservation between miR-1 and miR-206

Mus musculus miR-1 and miR-206

5'-UGGAAUGUAAAGAAGUAUGUAU-3' mmu-miR-1a-3p
5'-UGGAAUGUAAGGAAGUGUGUGG-3' mmu-miR-206-3p

Homo sapiens miR-1 and miR-206

5'-UGGAAUGUAAAGAAGUAUGUAU-3' hsa-miR-1
5'-UGGAAUGUAAGGAAGUGUGUGG-3' hsa-miR-206

c Timeline of myogenic differentiation experiments

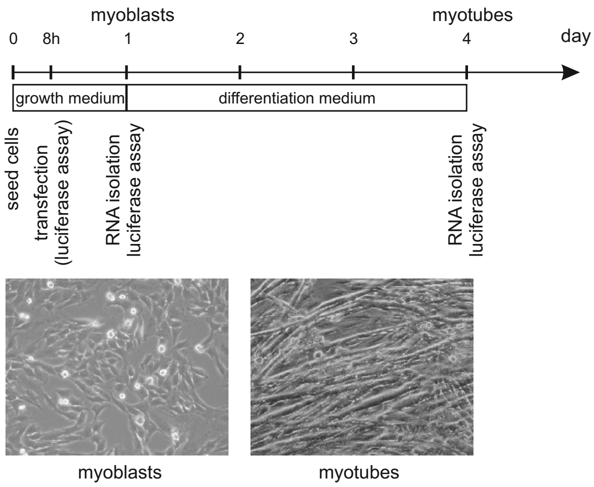

d BDNF protein in C2C12 cells

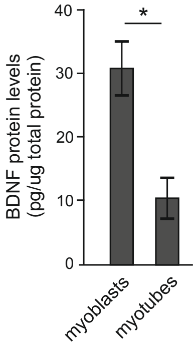

e miR expression in C2C12 cells

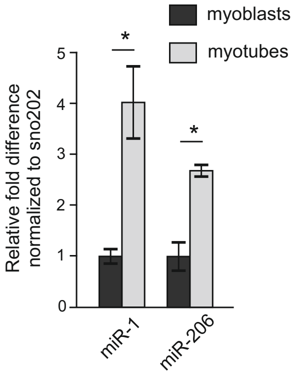

Supplement: Supplementary file 5 — Supplementary material 5 (PDF 1,436 kb) [file 18_2014_1628_MOESM5_ESM.pdf]
