# Supplementary material for: miR-1, miR-10b, miR-155, and miR-191 are novel regulators of BDNF
Source: Cell Mol Life Sci. 2014 May 8;71(22):4443–56. doi: 10.1007/s00018-014-1628-x (PMC4207943; doi:10.1007/s00018-014-1628-x)
Supplement: Supplementary file 6 — Supplementary material 6 (PDF 63 kb) [file 18_2014_1628_MOESM6_ESM.pdf]

**miR-1, miR-10b, miR-155 and miR-191 are novel regulators of BDNF**

Cellular and Molecular Life Sciences

Kärt Varendi, Anmol Kumar, Mari-Anne Härma and Jaan-Olle Andressoo*

Institute of Biotechnology, University of Helsinki, 00014, Finland

*To whom correspondence should be addressed. Tel. +358 50 358 1213; E-mail: jaan-olle.andressoo@helsinki.fi

**Online resource 5**

miR expression in different cells

| | **miR-1** | **miR-10b** | **miR-15a** | **miR-16** | **miR-30a** | **miR-30b** | **miR-155** | **miR-182** | **miR-191** | **miR-206** |
|---|---|---|---|---|---|---|---|---|---|---|
| **ARPE-19** | n.e | 0.000037 ± 0.000037 | 0.33 ± 0.19 | 2.66 ± 0.37 | 0.039 ± 0.023 | 0.14 ± 0.08 | 0.0042 ± 0.0019 | 0.073 ± 0.046 | 1 | 0.000098 ± 0.000067 |
| **U-87 MG** | 0.0012 ± 0.0003 | 0.047 ± 0.007 | 0.15 ± 0.01 | 10.5 ± 0.1 | 0.030 ± 0.003 | 0.59 ± 0.04 | 0.060 ± 0.001 | n.e | 1 | n.e |
| **HEK-293** | 0.00016 ± 0.00011 | 0.18 ± 0.05 | 0.024 ± 0.005 | 1.94 ± 0.06 | 0.013 ± 0.008 | 0.39 ± 0.28 | n.e | 0.017 ± 0.014 | 1 | 0.000048 ± 0.000001 |
| **C2C12 myoblasts** | 0.0092 ± 0.0022 | n.e | 0.0078 ± 0.0014 | 0.49 ± 0.11 | 0.0095 ± 0.0008 | 0.050 ± 0.006 | 0.0038 ± 0.0011 | 0.0025 ± 0.0002 | 0.24 ± 0.06 | 2.35 ± 0.65 |
| **C2C12 myotubes** | 0.030 ± 0.004 | n.e | 0.0073 ± 0.0009 | 0.89 ± 0.18 | 0.029 ± 0.001 | 0.095 ± 0.005 | 0.0052 ± 0.0009 | 0.0058 ± 0.0006 | 0.80 ± 0.17 | 6.31 ± 0.27 |
| **mouse hippocampus** | 0.0024 ± 0.0003 | n.e | n.t | n.t | n.t | n.t | 0.000071 ± 0.000050 | n.t | 0.21 ± 0.06 | n.t |
