# Supplementary material for: miR-1, miR-10b, miR-155, and miR-191 are novel regulators of BDNF
Source: Cell Mol Life Sci. 2014 May 8;71(22):4443–56. doi: 10.1007/s00018-014-1628-x (PMC4207943; doi:10.1007/s00018-014-1628-x)
Supplement: Supplementary file 7 — Supplementary material 7 (PDF 68 kb) [file 18_2014_1628_MOESM7_ESM.pdf]

miR-1, miR-10b, miR-155 and miR-191 are novel regulators of BDNF

Cellular and Molecular Life Sciences

Kärt Varendi, Anmol Kumar, Mari-Anne Härma and Jaan-Olle Andressoo*
Institute of Biotechnology, University of Helsinki, 00014, Finland
*To whom correspondence should be addressed. Tel. +358 50 358 1213; E-mail: jaan-olle.andressoo@helsinki.fi

Online resource 6

miR binding sites within SV40 polyA sequence predicted with PITA

microRNA	Position
mmu-miR-19b	26
mmu-miR-219	30
mmu-miR-33	45
mmu-miR-367	47
mmu-miR-92b	47
mmu-miR-301a	90
mmu-miR-301b	90
mmu-miR-367	115
mmu-miR-206	144
mmu-miR-125b-5p	159
mmu-miR-125a-5p	159
mmu-miR-490	160
mmu-miR-125b-5p	165
mmu-miR-125a-5p	165
mmu-miR-351	165
